# Near-Infrared Phosphorescent Hybrid Organic–Inorganic Perovskite with High-Contrast Dielectric and Third-Order Nonlinear Optical Switching Functionalities

**DOI:** 10.1021/acsami.1c20557

**Published:** 2021-12-30

**Authors:** Mirosław Ma̧czka, Andrzej Nowok, Jan K. Zarȩba, Dagmara Stefańska, Anna Ga̧gor, Monika Trzebiatowska, Adam Sieradzki

**Affiliations:** † Institute of Low Temperature and Structure Research, Polish Academy of Sciences, ul. Okólna 2, 50-422 Wrocław, Poland; # Department of Experimental Physics, Wrocław University of Science and Technology, Wybrzeże Wyspiańskiego 27, 50-370 Wrocław, Poland; ¶ Advanced Materials Engineering and Modeling Group, Faculty of Chemistry, Wrocław University of Science and Technology, Wybrzeże Wyspiańskiego 27, 50-370 Wrocław, Poland

**Keywords:** chromium, phosphorescence, dielectric switching, nonlinear optics, coordination polymers, third-harmonic generation switching

## Abstract

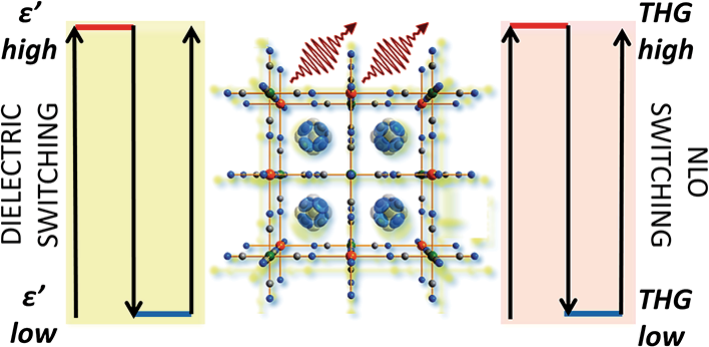

Hybrid organic–inorganic perovskites providing integrated functionalities for multimodal switching applications are widely sought-after materials for optoelectronics. Here, we embark on a study of a novel pyrrolidinium-based cyanide perovskite of formula (C_4_H_10_N)_2_KCr(CN)_6_, which displays thermally driven bimodal switching characteristics associated with an order–disorder phase transition. Dielectric switching combines two features important from an application standpoint: high permittivity contrast (Δε′ = 38.5) and very low dielectric losses. Third-order nonlinear optical switching takes advantage of third-harmonic generation (THG) bistability, thus far unprecedented for perovskites and coordination polymers. Structurally, (C_4_H_10_N)_2_KCr(CN)_6_ stands out as the first example of a three-dimensional stable perovskite among formate-, azide-, and cyanide-based metal–organic frameworks comprising large pyrrolidinium cations. Its stability, reflected also in robust switching characteristics, has been tracked down to the Cr^3+^ component, the ionic radius of which provides a large enough metal–cyanide cage for the pyrrolidinium cargo. While the presence of polar pyrrolidinium cations leads to excellent switchable dielectric properties, the presence of Cr^3+^ is also responsible for efficient phosphorescence, which is remarkably shifted to the near-infrared region (770 to 880 nm). The presence of Cr^3+^ was also found indispensable to the THG switching functionality. It is also found that a closely related cobalt-based analogue doped with Cr^3+^ ions displays distinct near-infrared phosphorescence as well. Thus, doping with Cr^3+^ ions is an effective strategy to introduce phosphorescence as an additional functional property into the family of cobalt-cyanide thermally switchable dielectrics.

## Introduction

Hybrid organic–inorganic perovskites (HOIPs) have generated tremendous attention due to their unique physicochemical properties, which can be tailored by prudent choice of organic and inorganic components.^1−5^ Much publicity has grown particularly around those materials that exhibit coexistence of two or more functional properties in one phase; hence, multiple functionalities can be harnessed to perform more than one task at the same time.^6−8^ Furthermore, coupling between different physicochemical properties may lead to emergent phenomena, thus enabling construction of new types of multifunctional devices.^9,10^ Prime examples of such multifunctionality in the broad perovskite family are three-dimensional (3D) lead halides exhibiting optoelectronic, photovoltaic, and nonlinear optical (NLO) properties.^2,3,5,11^ Multifunctionality, however, is not confined only to classic lead halide HOIPs; indeed, multifunctional properties were also reported for a number of 3D perovskites composed of metal centers linked by multiatomic ligands such as HCOO^–^, H_2_POO^–^, or N(CN)_2_^–^. For instance, some formates were shown to exhibit multiferroic properties,^12,13^ hypophosphites demonstrate coexistence of magnetic properties and photoluminescence (PL),^14,15^ and dicyanamides feature concomitant PL and second-order nonlinear optical (NLO) properties.^16,17^

One of the most widely investigated functionalities of 3D HOIPs is PL because this property is attractive for various applications, including LEDs, lasers, lighting, remote thermometers, etc.^10,15,18^ In lead perovskites, PL originates from recombination of excitons,^4,10^ but PL in HOIPs may also originate from transitions between electronic levels of metal centers^8,15^ or organic compounds.^4^ In the case of metal centers, Cr^3+^ is one of the most important PL species.^19−22^ PL associated with this transition metal cation depends strongly on the crystal field strength and changes from a broad band in the near-IR (NIR) region for the weak crystal field (^4^T_2g_→^4^A_2g_ fluorescence) to narrow in the red region (usually near 700 nm) for the Cr^3+^ in the strong crystal field (^2^E_g_→^4^A_2g_ phosphorescence).^19,20,23^ The phosphorescence of a ruby was utilized for construction of a solid-state red laser.^24^ Recently, there is growing interest in finding materials exhibiting efficient emission in the NIR region because such radiation has good penetration of organic matter and finds application in bioimaging, sensors, photovoltaics, optical communications, etc.^21,22,25,26^ Materials comprising Cr^3+^ are good candidates for NIR sources since they can be easily excited with the available blue LEDs.^21^ NIR fluorescence has been reported mainly for oxide inorganic materials,^19,21,22^ whereas NIR phosphorescence has been observed for some compounds comprising Cr^3+^ coordinated to nitrogen atoms^20,25,26^ or carbon atoms of cyanide ions.^27,28^ It is important to add that rational design of NIR phosphorescent materials with improved PL quantum yield requires maximizing the ligand field strength since a large energy gap between the ^2^E_g_ and ^4^T_2g_ states minimizes back intersystem crossing.^20^

Peculiar subtypes of multifunctional materials constitute switchable HOIPs, i.e., those whose physicochemical responses can be turned on (high) and off (low) by means of external stimuli such as temperature or pressure. By far, the most frequently targeted switching-based functionalities are dielectric switching and NLO switching. The former feature in HOIPs is highly relevant for modern technologies, i.e., sensors, switches, memory devices, signal processing, etc.^29,30^ It is typically achieved by the change of the mobility of polar ions between the static (low dielectric) and dynamic (high dielectric) states. Accordingly, significant dielectric anomalies require the presence of polar species, usually protonated amines, as well as necessitate their reversible ordering/disordering at a certain temperature or pressure. A coordination net that hosts organic cations plays a role but less obvious than organic guests. Regardless of their chemical identity, candidates for dielectric switching should be chemically stable and resistant to fatigue upon multiple switching cycles, and the dielectric response to an external stimulus should be fast.^31^ Weak frequency dependence of the dielectric permittivity is also a much coveted property.

Thus far, switchable dielectric properties were reported mainly for metal halides,^3,7,29,32−34^ but compounds with other linkers, for instance, perchlorate or thiocyanate, are also known.^30,34^ Most of the discovered compounds present isolated metal–ligand clusters, not beneficial for optical properties.^35^ 3D perovskite-type switchable dielectrics are still scarce and include methylhydrazinium lead bromide,^3^ dimethylammonium cadmium azide,^36,37^ and tetrapropylammonium cadmium dicynamide.^38^ However, the dielectric anomalies in these 3D perovskites are very weak and/or strongly frequency-dependent.^3,36−38^ In that context, the most promising family of 3D switchable perovskites constitutes cyanides of the general formula A_2_M^I^M^III^(CN)_6_ (A = organic cation; M^I^ = Na, K, or Rb; M^III^ = Co, Fe, or Cr). A step-like change of dielectric permittivity was reported for a few cobalt^39−45^ and iron^42,43,46,47^ cyanides, but a reversible change between on and off states by a thermal stimulus was demonstrated only for cobalt analogues comprising methylammonium (MA^+^) and iron analogues comprising MA^+^ and dimethylammonium (DMA^+^) cations.^39,47^ It is worth noting that we have discovered switchable properties controlled by temperature also for a nonperovskite cobalt cyanide network comprising pyrrolidinium (Pyr^+^) cations.^48^ Furthermore, we demonstrated for the first time that the change between on and off states in this compound may also be achieved by applying external pressure.^49^ Regarding chromium perovskite analogues, step-like dielectric anomalies were reported for two analogues only, i.e., DMA_2_KCr(CN)_6_ and MA_2_KCr(CN)_6_.^39,50^ In the latter case, the switching between on and off states was demonstrated but suffered from a poor stability of its ε′ upon prolonged cycling due to decomposition.^39^ The above inputs served as guideposts, stimulating our research in the direction of chromium cyanide-based perovskite materials that feature high-contrast dielectric switching but with much improved stability.

Switching of NLO properties in hybrid materials has become another leading theme these days. Although nonlinear optics offers a wide palette of second- and higher-order NLO phenomena that could be employed for that purpose, SHG is an absolutely dominant pathway by which the switching of NLO response is realized. Specifically, SHG switching is a second-order NLO outcome of temperature-induced transitions between crystal phases in which at least one of them is noncentrosymmetric.^34,51−59^ However, we see a great deal of untapped potential in the analogue parametric NLO process, namely, the third-harmonic generation (THG). By contrast to SHG, THG occurs in crystalline solids of any symmetry; hence, THG switching can be, in principle, performed even for all-centrosymmetric solids. Indeed, despite that fact, temperature-driven THG switching has never been reported for HOIPs, to the extent of our knowledge. What is even more perplexing, there were no attempts to employ THG for simple monitoring of structural phase transitions in HOIPs as well. Accordingly, the use of THG for perovskite material discovery is generally an uncharted territory that needs exploration.

The following contribution is devoted to the chromium cyanide network Pyr_2_KCr(CN)_6_, which was found to feature two disparate kinds of switching phenomena: dielectric switching and THG switching. While dielectric switching itself is not a new matter, the major advancement that we present is that we have overcome stability challenges of chromium cyanide networks by employing pyrrolidinium cations as an organic guest. By doing this, we have also managed to enhance the main merits of this dielectric switch, i.e., a very high contrast of the dielectric response (Δε′ = 38.5) and fast switching (minimum switching time = 1 min). We place a particular emphasis on the importance of the latter parameter, which is often overlooked but being of utmost importance for real-life applications.

We also demonstrate for the first time the THG switching for HOIP material, taking Pyr_2_KCr(CN)_6_ as a model. While this particular aspect of the present paper is much more of exploratory than applicational character, for the very first time, we provide proof of concept of efficient THG switching between two crystalline phases in HOIP material. By going beyond the former path of SHG to more exotic NLO phenomena, we paved the way to the development of alternative but still useful NLO switching schemes. Since the title compound is thus far an unknown material featuring phase transition behavior, characterization with X-ray crystallography, vibrational (IR and Raman) spectroscopies, and thermal techniques has been performed as well. Furthermore, we also targeted unusual phosphorescence properties of the title compound, adding up to its multifunctional character. It is worth noting that in order to better understand the relationship between the structure and optical properties of the cyanides comprising Pyr^+^ cations, we also report an optical study of the previously discovered nonperovskite cobalt analogue Pyr_2_KCo(CN)_6_ doped with Cr^3+^ ions.

## Experimental Section

### Synthesis

In order to grow Pyr_2_KCr(CN)_6_ crystals, 2.6 mL of pyrrolidine (30 mmol) dissolved in 20 mL of water was neutralized with about 3 mL of hydrochloric acid. Then, 5 mmol of K_3_Cr(CN)_6_ was dissolved in this solution on a hot plate at 50 °C under stirring for 2 h, the heating was switched off, and the solution was left at RT. After one week, yellow crystals were separated from the mother liquid and dried. The same method was used to grow Pyr_2_KCo(CN)_6_:Cr^3+^ crystals, but the mixture contained respective amounts of K_3_Cr(CN)_6_ and K_3_Co(CN)_6_. The comparison of their powder XRD patterns with the calculated ones based on the single-crystal data confirmed the phase purity of powdered samples (Figure S1 in the SI).

### X-ray Powder Diffraction

Powder XRD patterns were measured in the reflection mode on an X’Pert PRO X-ray diffraction system equipped with a PIXcel ultrafast line detector and Soller slits for Cu Kα_1_ radiation (λ = 1.54056 Å).

### Differential Scanning Calorimetry (DSC)

The heat flow was measured using a Mettler Toledo DSC–1 calorimeter with a high resolution of 0.4 μW. Nitrogen was used as a purging gas, and the sample weight was 22.50 mg. The calorimetric measurements were performed on heating and cooling cycles at rates of 1, 2, 5, 10, and 20 K min^–1^. The excess heat capacity associated with the phase transition was evaluated by subtracting from the data the baseline representing the variation in the absence of the phase transitions.

### Thermogravimetric Analysis (TGA)

A TGA study was performed in the temperature range of 300–1130 K using a PerkinElmer TGA 4000. The sample weight was ca. 11.8 mg, and the heating speed rate was 10 K/min. Pure nitrogen gas as the atmosphere was used.

### Single-Crystal X-ray Diffraction

The single-crystal X-ray diffraction data were collected at 295 and 100 K on an Xcalibur single-crystal diffractometer operating with graphite monochromated Mo Kα radiation (λ = 0.71073 Å) and a CCD Atlas camera. CrysAlisPro was used for data processing (Rigaku Oxford Diffraction, 2015). Absorption correction was applied by using multiscan methods in the SCALE3 ABSPACK algorithm. The room temperature (RT) structure was solved by direct methods and refined using the full-matrix least-squares method in the SHELXL2014/7 package.^60^ Owing to the disorder of Pyr^+^ cations, the hydrogen atoms were not introduced into the refinement. The low-symmetry structure was not solved due to the complex twinning. Figure S2 presents the reciprocal space reconstructions taken in the cubic and the low-symmetry phases. Crystal data, data collection, refinement results, and selected bond lengths are shown in Table S1.

### Raman and IR Measurements

Temperature-dependent Raman spectra were measured using a Renishaw inVia Raman spectrometer equipped with a confocal DM 2500 Leica optical microscope and a thermoelectrically cooled CCD as a detector. Excitation was performed using an argon laser (λ_exc_ = 488 nm). Temperature-dependent IR spectra were measured on a KBr pellet using a stand-alone Nicolet iN10 microscope. The spectral resolution in the Raman and IR studies was 2 cm^–1^. The temperature of the samples was controlled using a Linkam THMS 600 heating/freezing stage.

### Broadband Dielectric Spectroscopy (BDS)

The dielectric measurements were performed every 1 K using a Novocontrol Alpha impedance analyzer. The temperature (with stability higher than 0.1 K) was controlled by a Novocontrol Quatro system, by using a nitrogen gas cryostat. The single crystal with a crystallographic orientation perpendicular to the (001) plane had dimensions of 1.7 × 1.2 × 0.7 mm^3^. The silver paste was deposited on the surface as an electrode. The AC voltage with an amplitude of 1 V and frequency in the range 0.1 Hz to 1 MHz was applied across the sample. Each switching cycle was registered as time-dependent dielectric permittivity for 30 min at two constant temperatures. The temperature ramp between these temperatures was kept at 5 K min^–1^.

### Absorption and Photoluminescence Studies

For measurements of the absorption spectra, a Varian Cary 5E UV–vis–NIR spectrophotometer was used. Temperature-dependent PL spectra were recorded with a PMA-12 Hamamatsu photonic multichannel analyzer equipped with a BT-CCD linear image sensor, and 266 and 405 nm laser diodes were used as the excitation sources. The temperature of the samples during emission measurements was controlled using a Linkam THMS 600 heating/freezing stage. A temperature of 77 K was obtained using liquid nitrogen cooling, whereas for the phosphorescence measurement at 5 K, the sample was placed in an Oxford CF 1204 continuous-flow helium cryostat equipped with a temperature controller.

### THG Studies

Nonlinear optical studies were performed using a laser system consisting of a Coherent Astrella Ti:Sapphire regenerative amplifier providing 800 nm pulses (75 fs pulse duration, 1 kHz repetition rate) driving a wavelength-tunable TOPAS Prime optical parametric amplifier (OPA). The output of the OPA was set to 1350 nm. Prior to the measurements, the single crystals of Pyr_2_KCr(CN)_6_ were crushed with a spatula, fixed between microscope glass slides (forming tightly packed layers), sealed, and mounted to the sample holder. The laser beam was directed onto the sample at 45 degrees and was unfocused. Signal-collecting optics, mounted to the glass optical fiber, was placed perpendicularly to the plane of the sample (backscattering geometry), which was placed on a horizontally aligned holder. Scattered pumping radiation was suppressed with the use of a 750 nm shortpass dielectric filter (FESH0750, Thorlabs). A temperature-resolved THG study was performed using a 1350 nm laser beam, with the power limited to 290 mW and a spot area of 0.5 cm^2^. This experiment was performed for four heating/cooling rates (2, 5, 10, and 20 K min^–1^). The THG switching experiment was performed for three heating/cooling rates (2, 5, and 10 K min^–1^), and the same laser beam parameters were employed as for the TR-THG study. Temperature control of the sample was performed using a Linkam LTS420 heating/freezing stage. Excitation geometry, signal collection optics, and the sample preparation protocol were the same as for the THG switching experiment. The emission spectra collected in both experiments were recorded by an Ocean Optics Flame T spectrograph.

## Results and Discussion

### Single-Crystal X-ray Diffraction

**Figure 1:**
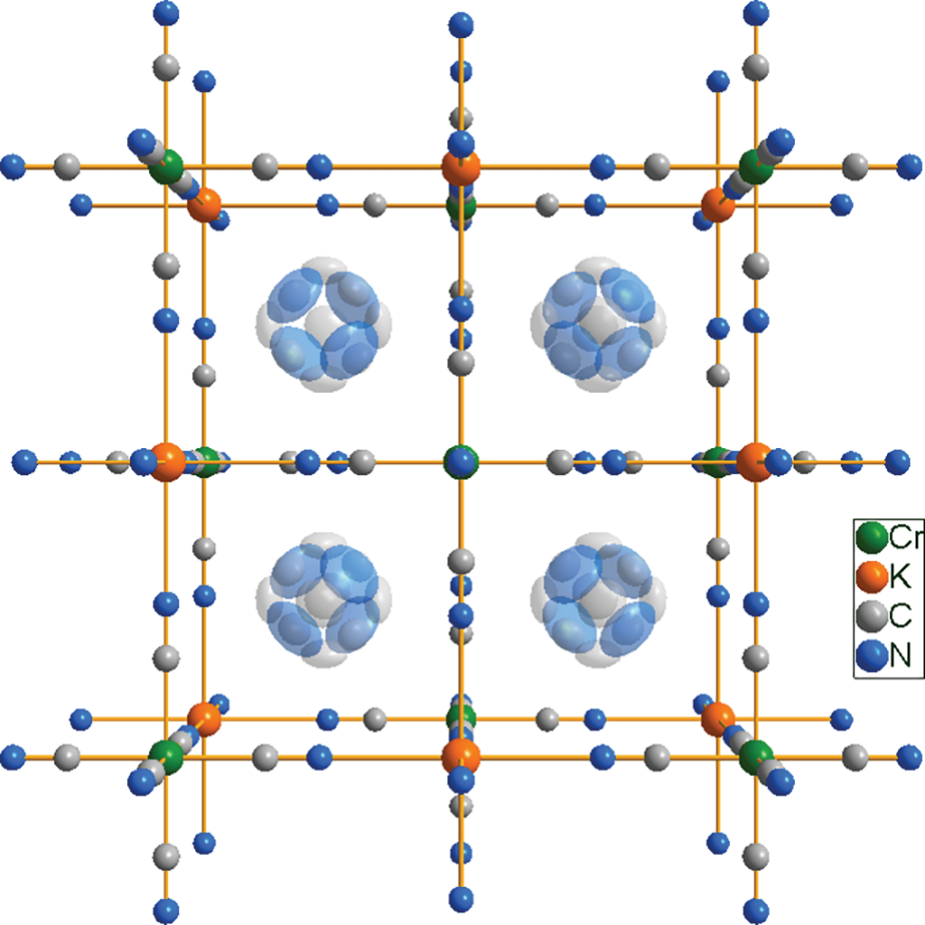
Crystal structure of Pyr_2_KCr(CN)_6_ in cubic phase I at 295 K. Pyr^+^ is massively disordered around the central point of the cage of 4̅3*m* symmetry.

Unexpectedly, Pyr_2_KCr(CN)_6_ crystallizes in the cubic *Fm*3̅*m* double perovskite structure (phase I, Table S1), isomorphic to the HT phases of A_2_KFe(CN)_6_ frameworks that crystallize with small ammonium cations such as formamidinium (FA^+^),^43^ MA^+^,^42^ or trimethylammonium (3MA^+^).^61^ It comprises a three-dimensional metal–cyanide scaffold and Pyr^+^ cations deployed in the structure cavities around the 4̅3*m* centers. Cr^3+^ ions are connected with six K^+^ neighbors by CN^–^ linkers (Cr^III^–C≡N–K). Both metal positions are coordinated by ideal CrC_6_ and KN_6_ octahedra of *O_h_* symmetry. The details of the crystal structure are presented in Figure 1. Due to the thermally activated rotations, the ammonium cations are disordered around the centers of cubic cages.

Owing to the ionic character of K–N bonds, the [KM^III^(CN)_6_] framework is prone to significant distortions resulting from the N–H···N hydrogen-bond (HB) interactions between ammonium cations and the framework. Depending on the size and shape of ammonium cations as well as the number and positions of proton donor centers, various low-symmetry polymorphs are stabilized already at RT. Both MA- and 3MA-based KFe^III^(CN)_6_ cyanides feature monoclinic *C*2/*c* RT symmetry,^61^ whereas the FA analogue crystallizes in the triclinic *P*1̅ space group.^43^ In Pyr_2_KCr(CN)_6_, the phase transition to the lower-symmetry polymorph II appears around 235 K. The diffraction patterns collected at 100 K unambiguously indicate the radical reduction of the crystal class, which is confirmed by a complex domain structure composed of at least 6 domain orientations. The ordering of Pyr^+^ at relatively low temperatures compared to other small ammonium cations results most likely from the ability of this cation to easy conformational changes between twisted and envelope forms, which hinder freezing of cationic motions and hydrogen-bond interactions.^62^

It is well-known that structural tunability of perovskites is limited by the size of the cavities that organic cations can accommodate. In the case of formate, azide, and cyanide frameworks, the largest organic cation used to date for synthesis of stable perovskite was tetramethylammonium (ionic size of 292 pm).^63,64^ For larger Pyr^+^ (320 pm)^65^ and thiazolium (ionic size of 320 pm),^64^ A_2_KCo(CN)_6_ analogues crystallize in nonperovskite structures containing large channels or cages.^44,48^ Thus, Pyr_2_KCr(CN)_6_ constitutes the first example of a stable perovskite in the family of formate–, azide–, and cyanide–metal frameworks comprising big Pyr^+^ cations. Inspection of crystallographic data for isostructural DMA_2_KM(CN)_6_ (M = Co, Fe, or Cr) cyanides shows that the M–C bonds (unit cell volume) increase from 1.894 and 1.924 Å (786.9 Å^3^) for the Co analogue^40^ to 1.932 and 1.944 Å (806.9 Å^3^) for the Fe analogue^61^ and 2.060 and 2.0805 Å (840.3 Å) for the Cr analogue^50^ due to the significantly larger ionic radius of Cr^3+^ (0.755 Å) compared to Fe^3+^ (0.69 Å) and Co^3+^ (0.685 Å).^66^ This example shows that employment of Cr^3+^ allows formation of significantly larger perovskite cages compared to the Co and Fe analogues. As a result, whereas the Pyr^+^ cation is too large to fit the perovskite cages in the Pyr_2_KM(CN)_6_ (M = Co or Fe) frameworks, it fits in the perovskite cages of the chromium analogue. Therefore, our results show that the incorporation of Cr^3+^ cations could be an effective way to synthesize cyanide-based perovskites accommodating some organic cations larger than 300 pm, such as thiazolium, tropylium, or isopropylammonium.

Worth adding is that the stability of a perovskite structure is often predicted based on the parameter called the tolerance factor (TF). The concept of the TF was extended to HOIPs by Kieslich et al.,^67^ and in the case of hybrid double perovskites A_2_M^I^M^III^X_6_, the TF can be calculated from the following equation:
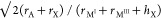
in which *r* denotes the effective radii of A, X, M^I^, or M^III^ and *h*_X_ is the effective height of the anion.^68^ For simple perovskites, cubic phases are usually found for TFs in the range of 0.9–1.0, whereas for TFs in the range of 0.8–0.9, perovskite networks are typically distorted, leading to lower-symmetry phases.^65,67^ For TFs higher than 1.0, the 3D perovskite structure becomes unstable, but there are some examples for 3D perovskites with TFs in the range of 1.0–1.1.^3,65,69^ The calculated TFs are 1.071, 1.070, and 1.059 for Pyr_2_KCo(CN)_6_, Pyr_2_KFe(CN)_6_, and Pyr_2_KCr(CN)_6_, respectively. Although for all compounds, the TF is significantly larger than 1.0, the smaller value of the TF for the Cr analogue, when compared to the Co and Fe counterparts, is consistent with the higher stability of the 3D double perovskite structure for this compound. Another point worth commenting on is that the phase transition temperature from the cubic disordered phase is much lower for Pyr_2_KCr(CN)_6_ (237.8 K on cooling) than for its MA analogue (447 K).^39^ A study of double perovskite cyanides comprising MA^+^ cations showed that the phase transition temperature decreases with an increasing TF, and the cubic phase would be stable below 298 K when the TF > 0.873.^68^ Therefore, the large increase in the phase transition temperature when going from Pyr_2_KCr(CN)_6_ to MA_2_KCr(CN)_6_ can be attributed to the large decrease in the TF for the latter compound (the TF of MA_2_KCr(CN)_6_ is 0.825).^68^ In the family of Pyr_2_M^I^Cr(CN)_6_ compounds, the decrease in the TF (increase in the phase transition temperature) could be realized by replacing smaller K^+^ cations with larger Rb^+^ or Cs^+^ cations.

### Raman and IR

Since our attempts to solve the low-temperature (LT) structure of Pyr_2_KCr(CN)_6_ were unsuccessful, we employed Raman and IR spectroscopic methods to obtain insight into the mechanism of the phase transition. Vibrational modes of Pyr_2_KCr(CN)_6_ can be subdivided into internal vibrations of Pyr^+^ cations and Cr(CN)_6_ units, translations of K^+^ and Cr(CN)_6_, and librations of Cr(CN)_6_. The RT phase (space group *Fm*3̅*m*) contains only one formula unit in the primitive cell. Since Pyr^+^ cations are disordered, we can only calculate the number of vibrational modes for the metal–cyanide framework (Table S2). Inspection of Table S2 shows that translations of K^+^ and Cr(CN)_6_ should contribute to two IR bands, whereas Cr(CN)_6_ librations (T_1g_ mode) should be silent. Factor group analysis also predicts that there should be four IR-active (4T_1u_), six Raman-active (2A_1g_+2E_g_+2T_2g_), and three silent (T_1g_+2T_2u_) internal modes of the Cr(CN)_6_ units. These modes can be classified as stretching and bending modes and described using the notation proposed by Jones^70^ (Table S2).

The most intense Raman and IR bands, observed at 2121 and 2116 cm^–1^, respectively (Table S3, Figure 3, and Figures S3–S5), can be assigned to CN^–^ stretching vibrations.^48,71^ Based on literature data reported for Prussian blue and related cyanides, we locate the remaining modes of the Cr(CN)_6_ units below 600 cm^–1^ (Table S3).^71,72^ The assignment of internal modes of Pyr^+^ cations, proposed in Table S3, is based on Raman and IR data reported for nonperovskite Pyr_2_KM(CN)_6_ analogues (M = Co or Fe).^48^

**Figure 2:**
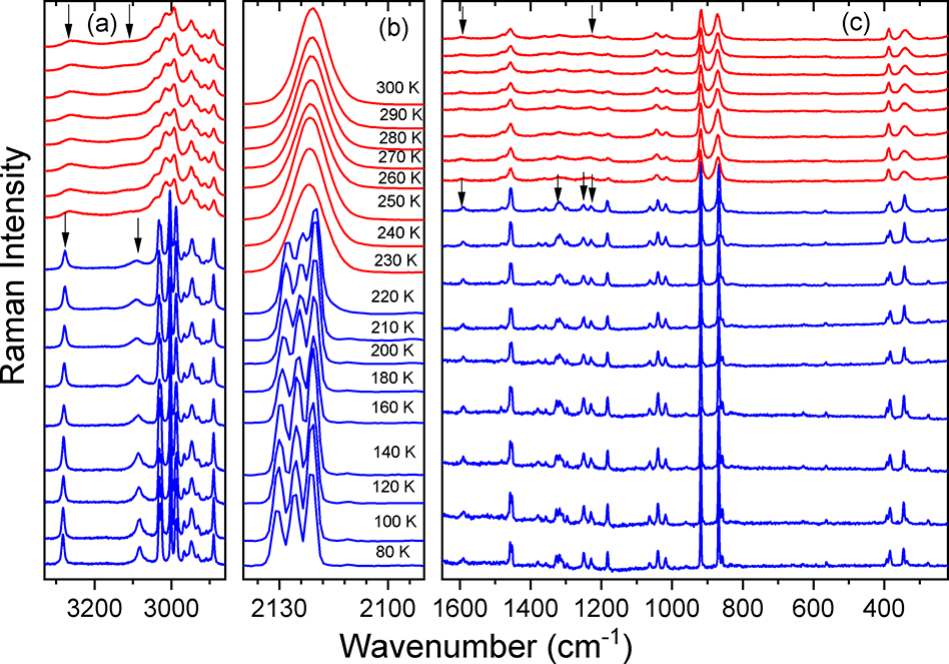
Temperature-dependent Raman spectra of Pyr_2_KCr(CN)_6_ in the (a) 3330–2860, (b) 2140–2090, and (c) 1650–220 cm^–1^ ranges. Arrows indicate modes related to vibrations of the NH_2_ group.

When temperature decreases, Raman and IR spectra exhibit drastic changes near 230 K, in line with the first-order character of the phase transition. First, the single νCN IR (Raman) band splits into a triplet at 2127+2122+2117 cm^–1^ (2131+2126+2121 cm^–1^) (Table S3, Figure 2, and Figures S3–S5). The fact that the Raman and IR modes are observed at different wavenumbers indicates that the LT phase is centrosymmetric. Furthermore, splitting of these bands into triplets is consistent with the complete lifting of degeneracy for the T_1u_ (ν_6_) IR-active mode and the E_g_ (ν_3_) Raman-active mode. The lifting of degeneracy and the presence of the inversion center are consistent with lowering of the LT phase symmetry to orthorhombic (point group *D*_2*h*_), monoclinic (point group *C*_2*h*_), or triclinic (point group *C_i_*). However, the fact that phase transition entropy is very large, comparable to those observed for FA and MA double perovskite cyanides exhibiting a phase transition to triclinic and monoclinic phases, respectively, suggests that symmetry of the LT phase is either monoclinic or triclinic. A decrease in the Cr(CN)_6_ site symmetry is further evidenced by splitting of the ν_4_, ν_10_, and ν_11_ Raman-active modes (Table S3). Note that in contrary to FA_2_KCo(CN)_6_ and FA_2_KFe(CN)_6_, which showed subtle changes of the CN-related Raman and IR bands at the phase transitions,^43^ the corresponding changes are very pronounced for Pyr_2_KCr(CN)_6_. This behavior indicates that the phase transition leads to a much stronger distortion of the chromium cyanide framework in Pyr_2_KCr(CN)_6_ than the metal–cyanide frameworks in the FA analogues. Second, both Raman and IR bands exhibit significant narrowing. This behavior is prevalent for a majority of Pyr^+^ bands, in particular the bands related to vibrations of the NH_2_ group (see Raman bands near 3260 and 3080 cm^–1^ in Figure 2 as well as IR bands near 3260, 3100, 1590, 1400, 1356, 1320, 1247, 1220, and 858–811 cm^–1^ in Figure S5). For instance, the full width at half maximum (FWHM) value of the Raman-active νNH_2_ mode at 3260 cm^–1^ (300 K value) changes from 66.9 cm^–1^ at 300 K to 7.3 cm^–1^ at 80 K. This behavior proves that the highly disordered Pyr^+^ cations in the high-temperature (HT) phase become well-ordered in the LT phase. Third, the NH_2_-related bands exhibit significant shifts. For instance, the IR-active νNH_2_ modes shift from 3261 and 3117 cm^–1^ at 300 K to 3282 and 3085 cm^–1^ at 80 K, while δNH_2_ shows softening by about 10 cm^–1^ (Table S3). This behavior points to significant rearrangement of HBs at the phase transition. Fourth, for a single Pyr^+^ cation, only six modes related to the NH_2_ group are expected (2 νNH_2_, δNH_2_, ωNH_2_, τNH_2_, and ρNH_2_ modes). The presence of four νNH_2_, two ωNH_2_, and two ρNH_2_ modes suggests either the presence of two unique Pyr^+^ cations in the LT phase or strong Davydov splitting.

### Thermal Behavior

**Figure 3:**
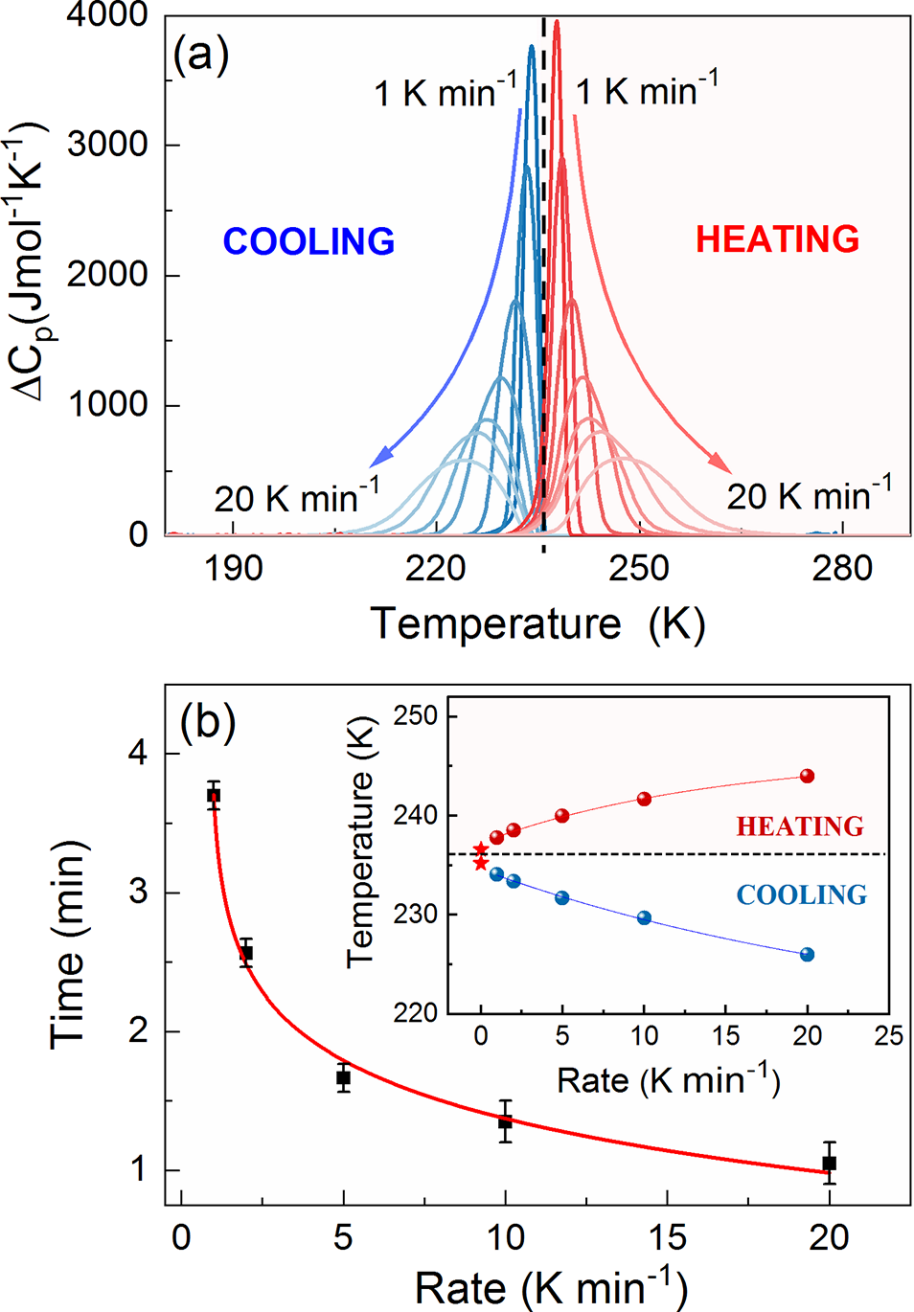
(a) Temperature dependences of Δ*C_p_* registered on heating and cooling at the rates in between 1 and 20 K min^–1^ for Pyr_2_KCr(CN)_6_. (b) Time required to overcome the temperature hysteresis between heating and cooling cycles at various temperature variation rates. The inset shows the phase transition temperature evolution when altering the scanning rate.

DSC has been performed to follow heat anomalies in Pyr_2_KCr(CN)_6_. As shown in Figure 3a, a single heat anomaly is detectable on each collected thermogram presented as a Δ*C_p_*(*T*), i.e., excess heat capacity calculated from the DSC data. Its symmetric shape indicates that the phase transition is of the first-order type. Very large changes of enthalpy (Δ*H* = 9.9 kJ mol^–1^) and entropy (Δ*S* = 42.4 kJ mol^–1^ K^–1^, Figure S6) both point to an order–disorder character of the phase transition. For an order–disorder transition, Δ*S* = *R* ln(*N*), where *R* is the gas constant and *N* is the ratio of the number of configurations in the disordered and ordered phases. The estimated *N* value associated with heat anomalies is about 15, indicating the high-order change at the phase transition temperature. One can expect that the disorder is related to the Pyr^+^ cations. It is worth noting that the Δ*S* value of Pyr_2_KCr(CN)_6_ perovskite is about three times larger than for nonperovskite Pyr_2_KM(CN)_6_ (M = Co or Fe) analogues that exhibit phase transition from the ordered LT *P*2_1_/*c* phase to the HT disordered *R*3̅*m* phase.^48^ Thus, DSC data indicate that the disorder of Pyr^+^ cations is significantly larger in the HT phase of perovskite Pyr_2_KCr(CN)_6_ compared to nonperovskite Pyr_2_KM(CN)_6_ analogues.

The anomaly occurs at 237.8 and 234.1 K on heating and cooling with a rate of 1 K min^–1^, respectively. Hence, there is a temperature hysteresis of 3.7 K between both cycles, which takes as long as 3.7 min to overcome. To reduce the switching time, one should consider (i) applying pressure or (ii) changing the temperature variation rate. The first option has already been tested on Pyr_2_KCo(CN)_6_, the nonperovskite cobalt analogue of the herein studied hybrid compound.^49^ Unfortunately, growing mechanical stresses in the material triggered several undesirable pressure-induced effects, such as the increase in hysteresis under compression or time requirement modification for the switching downward only. Therefore, we consider the latter solution in this article.

As shown in the inset of Figure 3b, the increase in the temperature variation rate modifies the phase transition temperature in a nonlinear way. Eventually, it occurs at 244 and 226 K during heating and cooling at 20 K min^–1^. Although the temperature hysteresis increases considerably up to 18 K, the time requirement to overcome it (, where Δ*T* is the temperature hysteresis value and d*T*/d*t* is the temperature variation rate) diminishes exponentially (Figure 3b). Therefore, the pace of the temperature-controlled structural transformation (and related dielectric and THG switching processes discussed in the further paragraphs) can be easily tuned by changing the temperature variation rate. Nevertheless, according to the performed calorimetric studies, the temperature hysteresis value is critical in regulating the switching time.

We also studied the thermal stability of Pyr_2_KCr(CN)_6_. Thermogravimetric data indicate that Pyr_2_KCr(CN)_6_ starts to decompose near 485 K (Figure S7). The weight loss between 485 and 645 K is about 49% and corresponds well to complete removal of pyrrolidinium cyanide (the calculated weight loss for decomposition of Pyrr_2_KCr(CN)_6_ into pyrrolidinium, potassium, and chromium cyanides is 50.2%). The stability of this double cyanide is larger than the stability of TMAO_2_KCo(CN)_6_ and TMAO_2_KFe(CN)_6_ (TMAO = (CH_3_)_3_NOH^+^), which start to decompose at 454 and 408 K, respectively.^72^ Its stability is, however, comparable to the stability of other chromium-based cyanides such as DMA_2_KCr(CN)_6_ (483 K)^50^ or MA_2_KCr(CN)_6_ (470 K).^39^

### Dielectric Studies

**Figure 4:**
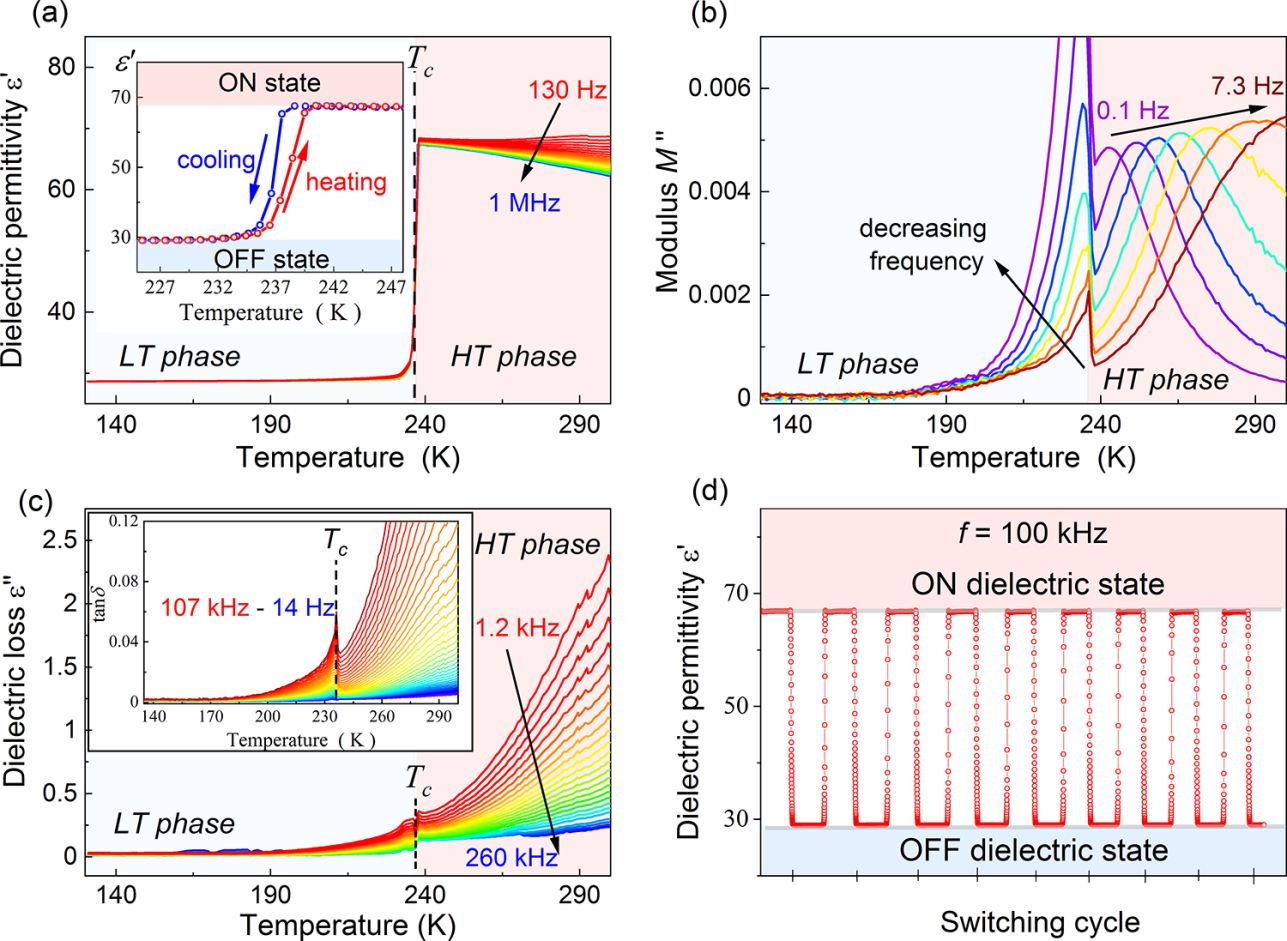
(a) Temperature dependence of ε′ registered in between 130 Hz and 1 MHz while heating. The inset shows a comparison of ε′(*T*) between heating and cooling cycles. (b) Temperature dependence of *M*″ registered in between 0.1 and 7.3 Hz while heating. (c) Thermal evolution of ε′′ registered on heating. The inset shows tan δ(*T*) dependence. (d) ε′ changes with the periodic variation of temperature between 245 and 225 K.

The temperature dependence of ε′, registered for a single crystal and presented in Figure 4a between 100 Hz and 1 MHz, reveals a typical image of the so-called dielectric switching. As indicated in the inset of Figure 4a, this process is fully reversible. Taking the LT phase as a starting point, ε′ almost does not vary with temperature up to 236 K, being close to 29, regardless of the selected frequency. This value can be associated with the low (off) dielectric state. During the structural transformation to the HT phase around 238 K, ε′ increases rapidly by 38.5 (133%) up to 67.5 independently of the applied frequency. As presented in Table 1, the ε′ value in the HT phase Pyr_2_KCr(CN)_6_ is higher relative to other hybrid organic–inorganic cyanides. Moreover, the change in ε′ at *T*_0_ (Δε′) is much larger compared to numerous metal–cyanide frameworks of both perovskite and nonperovskite architecture. For example, the nonperovskite pyrrolidinium-based Pyr_2_KM(CN)_6_ (M = Co or Fe) analogues are characterized by Δε′ values equal to only 6.6 and 7.4 along the [011] direction although they contain the same cage cation.^48,49^ Despite that large difference, the mechanism of the dielectric switching process in Pyr_2_KCr(CN)_6_ remains the same as for the pyrrolidinium-based analogues, i.e., relies on liberation of the Pyr^+^ motions during the order–disorder transition. This statement is evidenced by the imaginary *M*″ part of the complex dielectric modulus (Figure 4b). Specifically, its temperature dependence is characterized by a single relaxation process in the HT phase, whereas only a simple exponential decrease with lowering the temperature is detectable below *T*_0_. According to the literature, the source of the relaxation phenomena lies in the orientational freedom of the molecular cations in the cage-like lattice. Hence, the performed studies show that the activation of more motional possibilities through architecture changes to perovskite-like allows one to tune the dielectric switching parameters in hybrid compounds effectively.

**Table 1:** The Values of the Δε′ Parameter and ε′ in the Disordered HT Phase for Selected Hybrid Cyanides Measured at the Megahertz Range

	disordered HT phase				
material	SG	direction	ε′	Δε′	ref.
Pyr_2_KCr(CN)_6_	*Fm*3̅*m*	[001]	67.5	38.5	this work
Pyr_2_KCo(CN)_6_	*R*3̅*m*	[011]	23	6.6	(48)
Pyr_2_KFe(CN)_6_	*R*3̅*m*	[011]	24	7.4	(48)
DMA_2_KCr(CN)_6_	*P4*/*mnc*	[001]	26	23	(50)
		[110]	15	12	
DMA_2_KCo(CN)_6_	*P4*/*mnc*	[001]	19	15	(40)
		[110]	14	10	
MA_2_KCo(CN)_6_	*Fm*3̅*m*	[010]	47	30	(42)
		[101]	16	10	
		[111]	15	11	
HIm_2_KFe(CN)_6_	*R*3̅*m*	[12̅10]	27	21	(46)
		[0001]	7	1	
HIm_2_KCo(CN)_6_	*R*3̅*m*	[101]	22	16	(73)
		[001]	6	1	

To provide a more detailed explanation of this statement, one should consider factors affecting the Δε′ parameter for switchable dielectrics. First, it depends on the cage cation type, increasing with its dipole moment value. Second, the Δε′ value is direction-dependent (see Table 1), being highly influenced by the mechanism of the cage cation motion. Little to no change in ε′ at *T*_0_ is expected for those directions, for which the spatial rearrangement of the cage cations and their dipole moment vector is forbidden.^73^ Consequently, it is easier to encounter the desired direction with the highest Δε′ for compounds with a higher number of rearrangement possibilities of the cage cations. Indeed, such a situation is observed for Pyr_2_KCr(CN)_6_, the HT phase of which is more disordered concerning the pyrrolidinium cations compared to its nonperovskite analogues. Hence, to effectively tune the switchable features in hybrid compounds, one should search for the most disordered structures containing reorientable cage cations with the highest possible dipole moment value.

Figure 4c displays tan δ(*T*) and ε″(*T*) plots, which show a discontinuous anomaly at 236 K, connected with the structural transformation between LT and HT phases. However, contrary to *M*″, no relaxation processes are detectable in both representations. In general, these two quantities increase during heating because of the growing electrical conductivity contribution. Nevertheless, Pyr_2_KCr(CN)_6_ offers low dielectric losses in both phases (depicted in the inset of Figure 4c as low tan δ values), which is a highly desired feature for dielectrics from an application point of view.

Apart from the dielectric switching and low dielectric loss, Pyr_2_KCr(CN)_6_ offers another much coveted feature from an application point of view, which is excellent resistance to fatigue upon multiple switching cycles. To test this property, the temperature was varied between 245 and 225 K periodically so that the dielectric switching between off and on states could be triggered. As depicted in Figure 4d, the ε′ values of the off and on states were stable in time at the constant-temperature regime and remain unchanged upon numerous cycles. The fast time response of Pyr_2_KCr(CN)_6_ to temperature variation is also preserved. Consequently, the presented analysis allows us to classify Pyr_2_KCr(CN)_6_ as a low-loss switchable hybrid inorganic–organic compound, offering a significant change in ε′ at *T*_c_ and a good resistance to fatigue.

### Switching of THG Response

**Figure 5:**
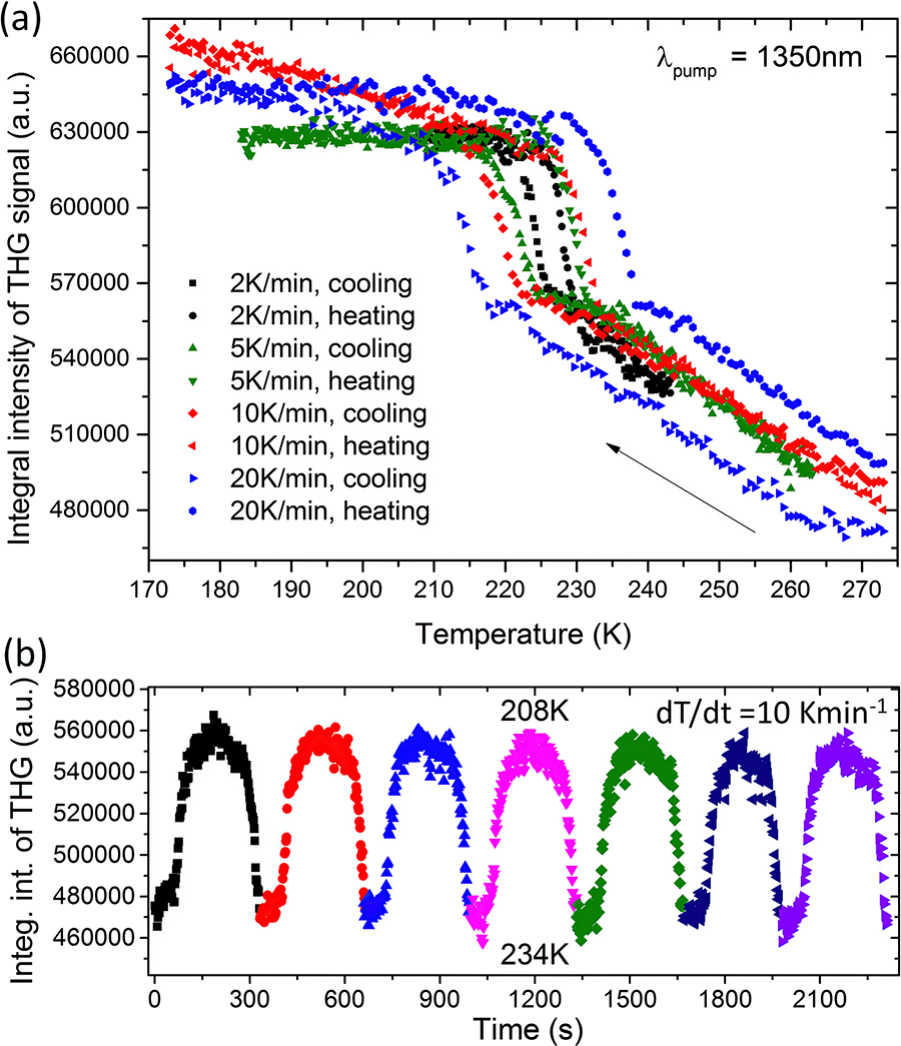
(a) Plots of integral intensities of the THG signal measured for four different heating/cooling rates: 2, 5, 10, and 20 K min^–1^. (b) Plot of integral intensities of the THG signal obtained during switching experiments between 234 and 208 K for a heating/cooling rate of 10 K min^–1^. Consecutive cycles are drawn with different colors.

Switchable features of Pyr_2_KCr(CN)_6_ are also demonstrated in its nonlinear optical properties. Interestingly, this compound shows no SHG, but the intensity of its THG signal centered at 450 nm significantly changes (10–12%) upon crossing the phase transition temperature. The first feature, i.e., no emission of SHG radiation at 675 nm under irradiation with 1350 nm femtosecond laser pulses, confirms the centrosymmetric space groups of both LT and HT phases. In turn, the latter property seems to be unique to Pyr_2_KCr(CN)_6_ since the analogous experiment for Pyr_2_KCo(CN)_6_ shows that the change in THG around *T*_0_ is basically insignificant (Figure S8). This feature indicates a particular role of the Cr^3+^ center in the investigated phase transition. Therefore, we used this opportunity to study unusual temperature-dependent THG response for Pyr_2_KCr(CN)_6_ systematically. To this end, we have monitored THG signal evolution during cooling and heating cycles for four different d*T*/d*t* rates (20, 10, 5, and 2 K min^–1^). As presented in Figure 5a, the width of the temperature hysteresis loop significantly widens as the heating rate increases, in line with calorimetric studies.

With the above dataset in hand, we proceeded to evaluate the THG switching performance. Boundary temperature points were chosen taking into account the hysteresis widths for each heating/cooling rate, in order to ensure complete conversion into the desired crystal phase. Results for the fastest d*T*/d*t* switching rate of 10 K min^–1^ are presented in Figure 5b, whereas those for rates of 2 and 5 K min^–1^ are shown in Figure S9. As seen in Figure 5b, switching between 234 and 208 K is reversible, and the THG signal keeps its intensity despite consecutive heating and cooling cycles. Upward and downward drifts in THG responses, noticeable over several consecutive cycles for the slowest d*T*/d*t* rates of 2 and 5 K min^–1^, are not sample-related but are due to long-term laser oscillations (Figure S9).

Further, by taking minimum and maximum values of integral THG intensities obtained at boundary temperatures, one can calculate the mean contrast of the THG switch. It turns out that the obtained mean contrast ratio is 1.2:1. If one compares this value with values of contrast reported for the most prevalent SHG-on–SHG-off quadratic NLO switches, the obtained value may seem not really high. For instance, of the highest contrast ratios (74:1 at around 328 K) was obtained by Zhang et al. for a material of the formula (MeNHEt_2_)[Cd(SCN)_3_].^74^ Clearly, a high contrast of SHG-on–SHG-off quadratic NLO switches can hardly be beaten, as this kind of NLO switching essentially operates against zero (nearly zero) background.

As indicated in previous sections, Pyr_2_KCr(CN)_6_ is the first HOIP material to feature THG switching, to the best of our knowledge. For this reason, it is not possible to provide apple-to-apple comparisons with similar materials. However, if we reach out to different material classes, a handful of examples of THG switching can be found in the literature. For instance, THG switching has been demonstrated for all-inorganic materials such as dichalcogenides and chalcogenide Ge–Sb–Te alloys (glasses). One example of a dichalcogenide THG switch is Sb_2_S_3_ sandwiched in between SiO_2_ layers. This composite, embedded in a Fabry–Pérot cavity, featured a THG contrast of 100:1 upon a phase change.^75^ A conceptually similar device employing Ge_2_Sb_2_Te_5_ revealed a difference of three orders of magnitude in THG intensity due to the phase transition.^76^ What needs stressing is that the phase transition occurs between crystalline and amorphous phases in these cases and explains why contrast ratios are so high for these compounds—THG for amorphous phases is practically suppressed. In this context it becomes clear why the THG contrast ratio for the title material is not as high as for the above examples—both phases of Pyr_2_KCr(CN)_6_ are crystalline.

### Linear Optical Properties

The diffuse reflectance spectrum of Pyr_2_KCr(CN)_6_ shows broad bands at 314.7 (31,776 cm^–1^), 402.2 (24,863 cm^–1^), and 528.7 nm (18,914 cm^–1^) (Figure S10) that can be assigned to electron transitions from the ^4^A_2g_ ground level to ^4^T_1g_, ^4^T_2g_, and ^2^T_2g_ excited states of Cr^3+^, respectively.^77^ It is worth noting that the positions of these bands are strongly blueshifted compared to typical absorption bands of Cr^3+^ coordinated to oxygen ions.^78−81^ The observed blueshift results from a strong crystal field around chromium ions, which are coordinated by the cyanide groups (Cr–C≡N–K). Due to the low absorption cross section, the zero-phonon line of ^2^E_g_ has not been detected. Pyr_2_KCo(CN)_6_ samples doped with Cr^3+^ show broad bands at 317.3 (31,516 cm^–1^) and 400.4 nm (24,975 cm^–1^) that can be attributed to electron transitions from the ^1^A_1g_ ground level to ^1^T_1g_ and ^3^T_1g_ excited states of Co^3+^, respectively.^82^ These bands overlap with weaker ^4^A_2g_→^4^T_1g_ and ^4^A_2g_→^4^T_2g_ bands related to Cr^3+^.

**Figure 6:**
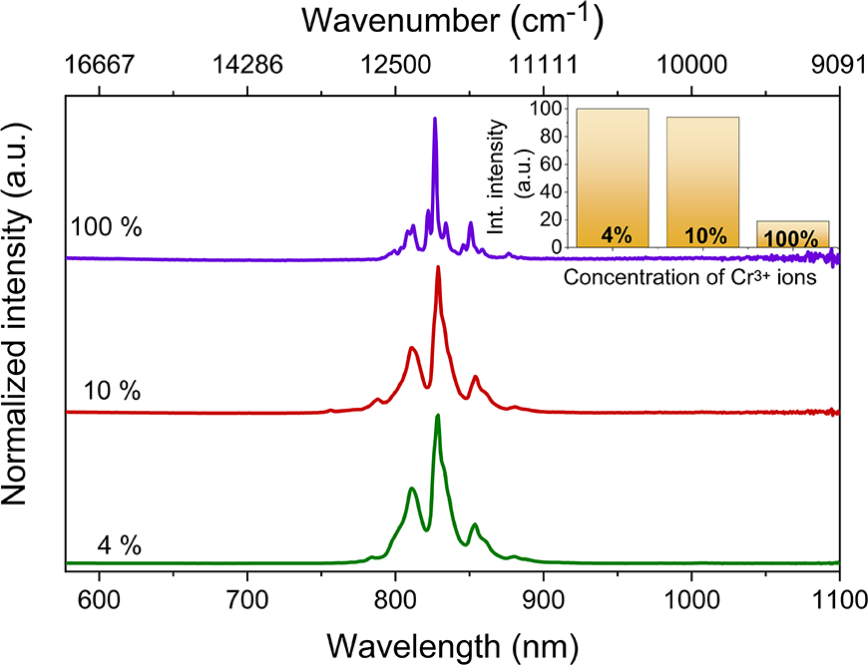
Normalized PL spectra of Pyr_2_KCr(CN)_6_ and Pyr_2_KCo(CN)_6_ doped with 4 and 10% Cr^3+^ ions at 77 K. The inset shows the influence of Cr^3+^ ion concentration on the PL intensity.

As can be seen in Figure 6, the emission bands of Cr^3+^ ions in the investigated compounds can be assigned to spin-forbidden ^2^E_g_→^4^A_2g_ transitions. However, a very rich band structure makes the explanation of the individual bands’ origin not trivial. Based on the literature data on inorganic cyanides comprising Cr^3+^^77,83,84^ as well as the phosphorescence spectra of Pyr_2_KCr(CN)_6_ measured at 5 K (Figure S11), the band at around 12,438 cm^–1^ (804 nm) was assigned to the 0–0 phonon line called R_1_. In the case of the Pyr_2_KCo(CN)_6_:Cr^3+^ samples, this line is observed near 12,500 cm^–1^ (800 nm). It is important to emphasize that the observed phosphorescence is very strongly redshifted compared to the phosphorescence of oxide materials (typically near 700 nm) or even compared to molecular Cr^3+^ complexes (λ_em_ below 777 nm)^19,20^ due to the strong crystal field around the chromium ions. In addition to the R_1_ line, many narrow bands are observed for Pyr_2_KCr(CN)_6_ on the low-energy side of this line. A majority of these bands can be assigned to vibronic transitions, but some of them may probably be attributed to N lines since the presence of N lines has been observed for many compounds heavily doped with Cr^3+^ and classified as the emission of chromium pairs (Cr^3+^–Cr^3+^).^80,81,85^ It can be noticed that the narrow bands form three major groups visible in the 808–812, 821–834, and 845–858 nm ranges (Figure 6). Three very similar groups of bands, although much less resolved, are also observed for the Pyr_2_KCo(CN)_6_:Cr^3+^ samples (Figure 6 and Figures S12–S14). In the case of inorganic cyanides, the most intense vibronic bands, shifted by less than 460 cm^–1^ with respect to the 0–0 phonon line, were attributed to IR-active fundamental vibrations of a metal–cyanide framework.^83^ On the other hand, weaker vibronic bands shifted by more than 500 cm^–1^ could be explained as arising from vibrational combination modes. By analogy with inorganic cyanides, we can attribute the three mentioned above groups of bands, shifted by about 60–130, 270–450, and 600–800 cm^–1^ with respect to the R_1_ line, to the ν_9_ and ν_13_ fundamental modes, ν_7_, ν_8_, and ν_12_ fundamental modes, and combination modes, respectively, of the Cr(CN)_6_ units.^83^ However, due to lack of temperature-dependent IR data in the far-infrared range, a more detailed assignment of vibronic bands could not be proposed.

The inset in Figure 6 presents the effect of Cr^3+^ concentration on the PL intensity. The highest intensity is observed for the sample containing the lowest concentration of Cr^3+^ ions, while the PL intensity of the Pyr_2_KCr(CN)_6_ sample is about 5 times lower. This effect can be related to the concentration quenching processes.

Crystal field (*Dq*) and Racah (*B*) parameters were calculated for the investigated compounds from the diffuse reflectance absorption and emission spectra. The value of the *B* parameter was found by setting the determinant (where *T*_F_ is the energy of the ^4^T_1_(F) band) defined below equal to 0.
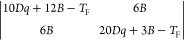


The transition energies and the calculated values of crystal field parameters are presented in Table 2. The *Dq*/*B* parameter is equal to 3.9 for Pyr_2_KCr(CN)_6_. The value of *Dq*/*B* higher than 2.3 confirms a strong crystal field in the immediate vicinity of Cr^3+^ ions. Obtained crystal field parameters and transition energies are similar to the values reported for K_3_[Cr_*x*_Co_1–*x*_(CN)_6_].^77^ However, the calculated *Dq* and Racah parameters are much higher than for recently reported formate perovskites.^78−80^

**Table 2:** Transition Energies, the Ligand Field Strength Parameter *Dq*, and the Racah Parameter for Pyr_2_KCr(CN)_6_

transition							
^4^A_2g_→^4^T_2g_	^4^A_2g_→^4^T_1g_	^4^A_2g_→E_g_	*Dq*	*B*	*C*	*Dq*/*B*	*C*/*B*
24,863	31,776	12,438	2486	637	2524	3.9	3.96

**Figure 7:**
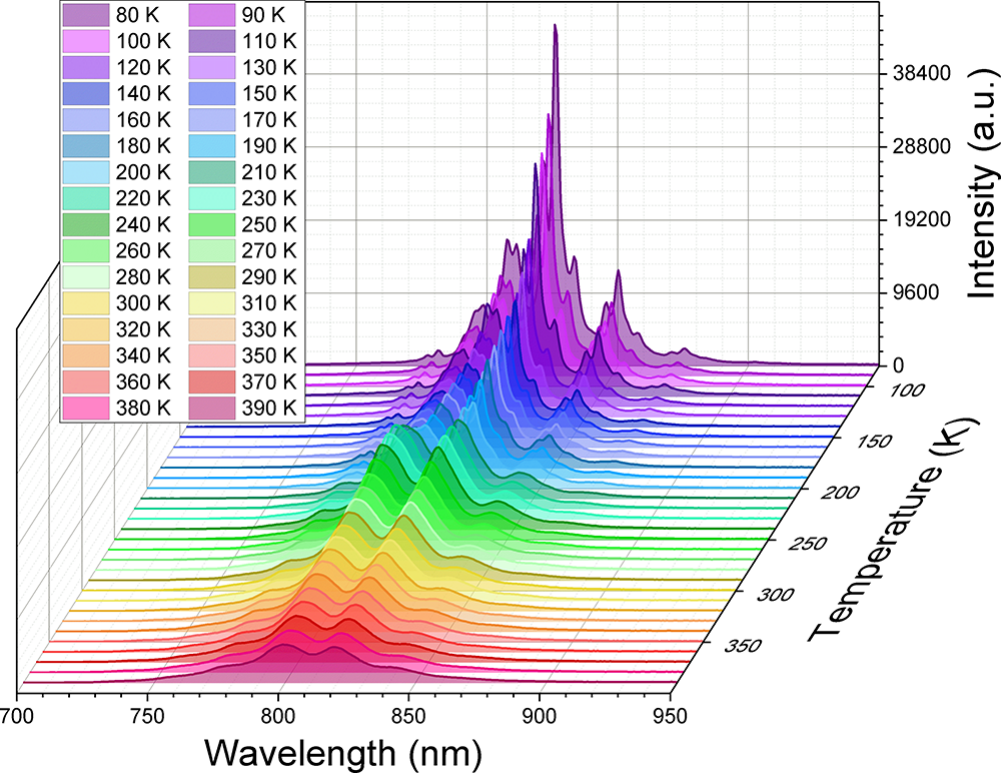
Temperature-dependent PL spectra of Pyr_2_KCr(CN)_6_ under 266 nm excitation.

Temperature-dependent emission spectra of Pyr_2_KCo(CN)_6_ doped with 4% Cr^3+^ ions are presented in Figure S13. Under 266 nm excitation, the sample shows strong PL bands related to the spin-forbidden transition of Cr^3+^ ions and low-intensity bands related to the π⃗π* ligand transition. The latter bands are observed in the 400–700 nm range (see the inset in Figure S13). Due to the fact that the host emission overlaps with ^4^T_2g_ and ^2^T_2g_ absorption bands of Cr^3+^ ions, the probability of the reabsorption process increases with the dopant content. It can be seen that the PL related to the spin-forbidden transition of Cr^3+^ ions is stable with the temperature both for Pyr_2_KCo(CN)_6_:Cr^3+^ and Pyr_2_KCr(CN)_6_ (Figure 7 and Figures S13 and S14). However, broadening of the PL bands with heating the samples is observed. Temperature-dependent studies of Pyr_2_KCr(CN)_6_ also show an increase in PL intensity as well as splitting of bands near 220–230 K, which can be related to the phase transition occurring near 235 K.

## Conclusions

The foregoing results demonstrate an effective strategy to obtain a temperature-switchable material with divergent, third-order nonlinear optical and dielectric switching modes. The application of Cr^3+^ as metal nodes for construction of a metal–cyanide network affords interstitial sites big enough to accommodate large pyrrolidinium organic cations. As a result, Pyr_2_KCr(CN)_6_ distinguishes itself in terms of temperature-induced switching stability, much improved compared to Cr^3+^ analogues comprising other organic cations or strained analogue materials comprising smaller Co^3+^ or Fe^3+^ ions. The other beneficial outcome for temperature switching capability is that Pyr^+^ cations experience enhanced reorientational freedom in the HT phase, making dielectric switching possible with a huge change in the dielectric permittivity value (Δε′ = 38.5) during the order–disorder structural transition at around 238 K.

Despite the fact that THG occurs in any crystalline solid, THG switching has never been reported for HOIP materials. In this contribution, we have demonstrated proof of concept of the reversible temperature switching of THG response, as well as for the first applied THG spectroscopy to track the phase transition in general. We thus expect a new field of THG switching in HOIPs to emerge soon.

We also demonstrate that Pyr_2_KCr(CN)_6_ exhibits strongly redshifted NIR phosphorescence, extending from 770 to 880 nm. The unprecedentedly large redshift can be attributed to the very large crystal field strength (*Dq*/*B* = 3.9). Nonperovskite Pyr_2_KCo(CN)_6_ doped with Cr^3+^ ions also shows NIR phosphorescence. Thus, doping with Cr^3+^ ions is an effective way to introduce PL as an additional functional property to the family of cobalt-cyanide switchable dielectrics. Furthermore, NIR PL can be tuned by alloying of chromium and cobalt cyanides as well as use of various alkali metal and organic cations.

We also envision that Cr^3+^-based extension of cyanide networks will make synthesis of 3D perovskite cyanide networks accommodating very large organic cations such as thiazolium, tropylium, and the like possible. Therefore, our results pave the way to new cyanide-based perovskites and show how to merge and effectively tune the switchable dielectric, THG, and NIR PL properties much desired in sensing, bioimaging, and optoelectronic applications.

## Supporting Information Available

Crystallographic data, vibrational selection rules, Raman and IR wavenumbers and assignment of modes, PXRD patterns, reciprocal space reconstruction, change in entropy at the phase transition, Raman and IR spectra, spectra of THG signals, diffuse reflectance spectra, PL plots, and temperature-dependent decay times (PDF)
CIF file for the structure at RT (CIF)

## Supplementary Material

am1c20557_si_001.pdf

am1c20557_si_002.cif
