# Supplementary material for: Near-Infrared Phosphorescent Hybrid Organic–Inorganic Perovskite with High-Contrast Dielectric and Third-Order Nonlinear Optical Switching Functionalities
Source: ACS Appl Mater Interfaces. 2021 Dec 30;14(1):1460–71. doi: 10.1021/acsami.1c20557 (PMC8762641; doi:10.1021/acsami.1c20557)
Supplement: Supplementary file 1 — am1c20557_si_001.pdf [file am1c20557_si_001.pdf]

Supporting Information for:

Near-Infrared Phosphorescent Hybrid Organic-Inorganic Perovskite with High-Contrast Dielectric and Third-Order Nonlinear Optical Switching Functionalities

Mirosław Mączka,,† Andrzej Nowok, # Jan K. Zaręba,*,¶ Dagmara Stefańska,† Anna Gągor,† Monika Trzebiatowska† and Adam Sieradzki#*

†Institute of Low Temperature and Structure Research, Polish Academy of Sciences, ul. Okólna 2, 50-422 Wrocław, Poland

#Department of Experimental Physics, Wrocław University of Science and Technology, Wybrzeże Wyspiańskiego 27, 50-370, Wrocław, Poland

¶Advanced Materials Engineering and Modeling Group, Faculty of Chemistry, Wrocław University of Science and Technology, Wybrzeże Wyspiańskiego 27, 50-370, Wrocław, Poland

*e-mail: m.maczka@intibs.pl (Mirosław Mączka)
*e-mail: jan.zareba@pwr.edu.pl (Jan K. Zaręba)

Table S1. Experimental details

	Phase **I**
Crystal data	
Chemical formula	$C_{14}H_{20}CrKN_8$
M_r	391.48
Crystal system, space group	Cubic, $Fm\bar{3}m$
Temperature (K)	295
a (Å)	12.3599 (4)
V (Å^3)	1888.19 (18)
Z	4
Radiation type	Mo $K\alpha$
μ (mm^{-1})	0.84
Crystal size (mm)	0.15 × 0.08 × 0.06
Data collection	
T_{min}, T_{max}	0.947, 1.000
No. of measured, independent and observed [$I > 2\sigma(I)$] reflections	1806, 160, 146
R_{int}	0.017
$(\sin \theta/\lambda)_{max}$ (Å^{-1})	0.681
Refinement	
$R[F^2 > 2\sigma(F^2)]$, $wR(F^2)$, S	0.032, 0.106, 1.08
No. of reflections	160
No. of parameters	19
H-atom treatment	H-atom parameters not defined
$\Delta\rho_{max}$, $\Delta\rho_{min}$ (e Å^{-3})	0.16, -0.31

Computer programs: *CrysAlis PRO* 1.171.38.41 (Rigaku OD, 2015), *SHELXT* 2014/5 (Sheldrick, 2014), *SHELXL2018*/3 (Sheldrick, 2018).

Table S2. Normal modes of $Cr(CN)_6$ and K^+ in the RT phase. Number of modes for Pyr^+ are not provided due to disorder of these cations. The IR-active modes are marked in red, the Raman-active - in blue and the silent ones - in black.

Ion	Vibration	Free ion	Site symmetry	Factor group
**$Cr(CN)_6^-$**		**O_h**	**O_h**	**O_h**
	νCN			
	ν_1	A_{1g}	A_{1g}	A_{1g}
	ν_3	E_g	E_g	E_g
	ν_6	T_{1u}	T_{1u}	T_{1u}
	νCr-C			
	ν_2	A_{1g}	A_{1g}	A_{1g}
	ν_4	E_g	E_g	E_g
	ν_8	T_{1u}	T_{1u}	T_{1u}
	δCrCN			
	ν_5	T_{1g}	T_{1g}	T_{1g}
	ν_7	T_{1u}	T_{1u}	T_{1u}
	ν_{10}	T_{2g}	T_{2g}	T_{2g}
	ν_{12}	T_{2u}	T_{2u}	T_{2u}
	δCCrC			
	ν_9	T_{1u}	T_{1u}	T_{1u}
	ν_{11}	T_{2g}	T_{2g}	T_{2g}
	ν_{13}	T_{2u}	T_{2u}	T_{2u}
	T’		T_{1u}	T_{1u}
	L		T_{1g}	T_{1g}
**K^+**			**O_h**	**O_h**
			T_{1u}	T_{1u}

Table S3. The observed Raman and IR modes (in cm^{-1}) of $Pyr_2KCr(CN)_6$ and the proposed assignment.

IR		Raman		Assignment
80 K	300 K	80 K	300 K	
3282s	3261s	3282m	3260w,b	νNH_2
3156m+3140m				νNH_2
3085s	3117s	3083w	3120vw,b	νNH_2
3032w+3021w		3031m+3026m	3040w	$\nu_{as}CH_2$
2999w		3001m	3013m	$\nu_{as}CH_2$
2988w+2985w	2986vw	2989m+2985m	2992m	$\nu_{as}CH_2$
				$\nu_{as}CH_2$
2964w	2960vw	2966w		$\nu_s CH_2$
		2948w	2948w	$\nu_s CH_2$
2892w	2890w	2932vw	2934vw	$\nu_s CH_2$
		2909w	2912vw	$\nu_s CH_2$
2891w	2890w	2890w	2891w	$\nu_s CH_2$
		2131s	2121vs	$\nu CN(\nu_1)$
		2126s+2121vs		$\nu CN(\nu_3)$
2127w+2122w+2117m	2116m			$\nu CN(\nu_6)$
2110w		2111vw		overtone
2074w	2075vw		2075vw	overtone
1587s	1597s	1590vw	1594vw	δNH_2
1514w	1521vw			δCH_2
1463m	1465sh	1477vw	1475vw	δCH_2
1459m+1455m	1460s	1457w+1452w	1457w	δCH_2
1403m				ωNH_2
1377s	1372s	1379vw	1360vw	ωCH_2
1356m				ωCH_2
1328s	1338w	1328w		ωNH_2
1319w+1313m	1321w	1318w+1311vw	1322vw	τCH_2
1292m	1298w	1297vw	1300vw	τCH_2
1258vw	1256vw			τCH_2
1247m	1247m	1250w		τNH_2
1229w+1223w		1228w	1229vw	τNH_2
1177w	1180w	1183w	1181vw	τCH_2
1061w	1060vw	1063w	1064sh	Ring
	1045w	1040w	1044w	Ring
1017s	1017m	1019w	1015vw	Ring ρCH_2
962w	971w,b			ρCH_2
920w+912w	918w	919m	918m	Ring ρCH_2
868w	870w	868m	872m	Ring

858w		858w		Ring
837w				ρNH_2
811w				ρNH_2
668vw	668vw	657vw	667vw	Ring
630w+607w	622w,b	630vw	623vw	Ring
564w	568w,b	564vw	567vw	Ring
		393w+384w	387w	$\delta CrCN(\nu_{10})$
		345w+336vw	341w	$\nu CrC(\nu_4)$+T'Pyr^+
		276vw	279vw	T'Pyr^+
		185m+151s	151m	$\delta CCrC(\nu_{11})$

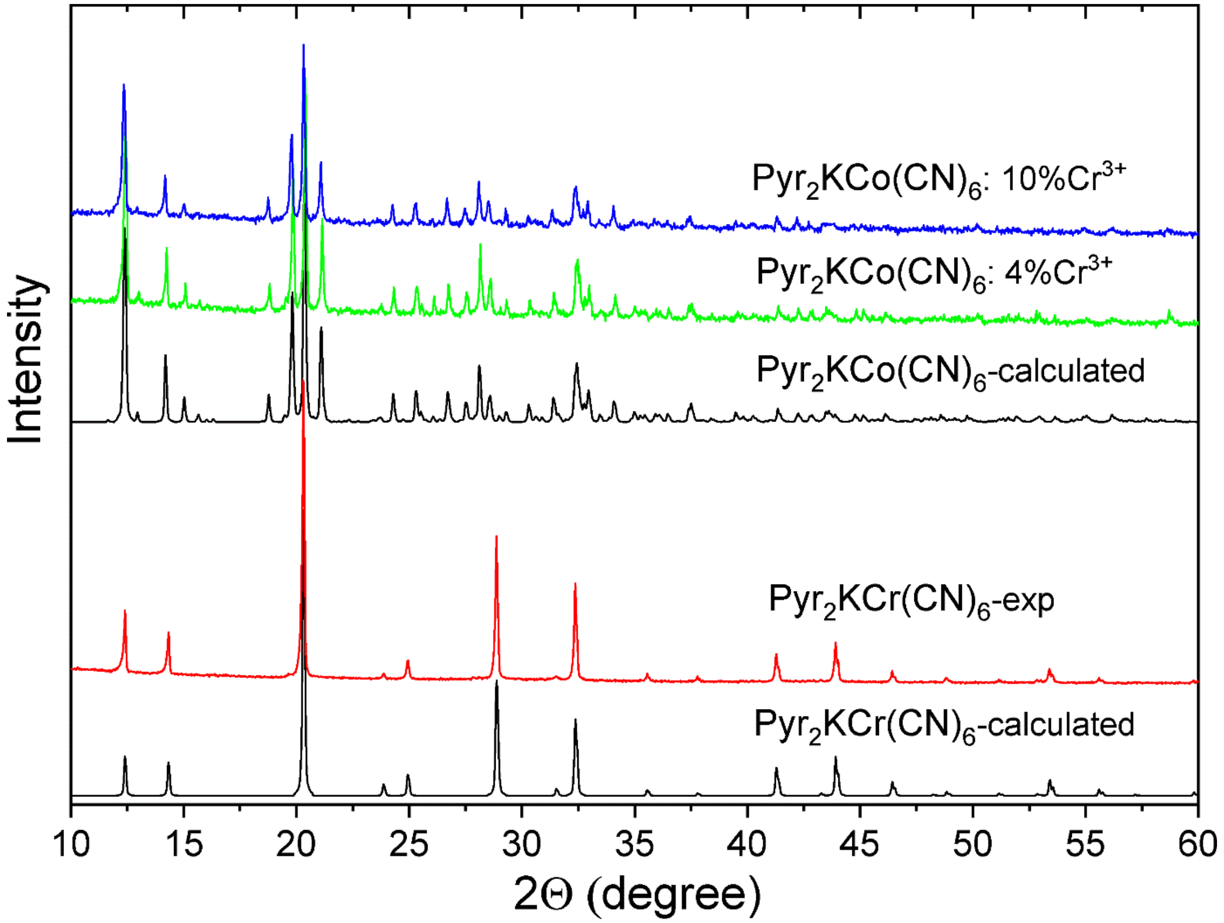

Figure S1. Powder XRD pattern for $Pyr_2KCr(CN)_6$ and $Pyr_2KCo(CN)_6$:Cr^{3+} together with the calculated ones based on the RT single crystal structures.

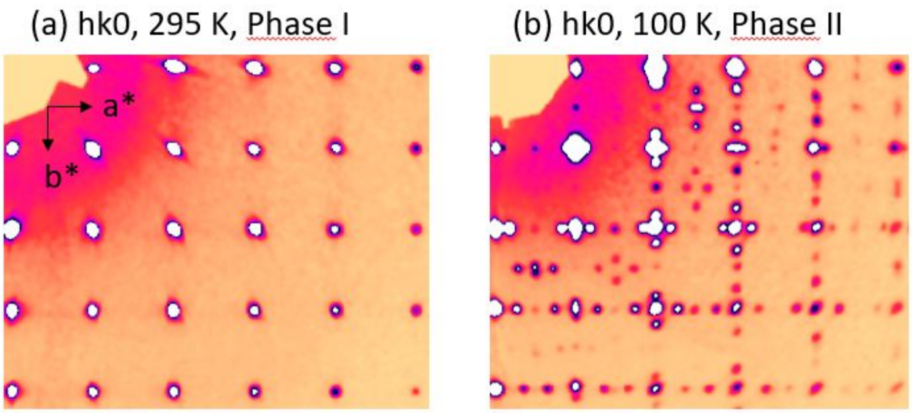

Figure S2. Reciprocal space reconstructions (hk0 layers) of $Pyrr_2KCr(CN)_6$ at (a) 295 K, cubic Phase **I** and (b) 100 K, low-temperature Phase **II**. A complex twin structure is formed due to the large symmetry lowering.

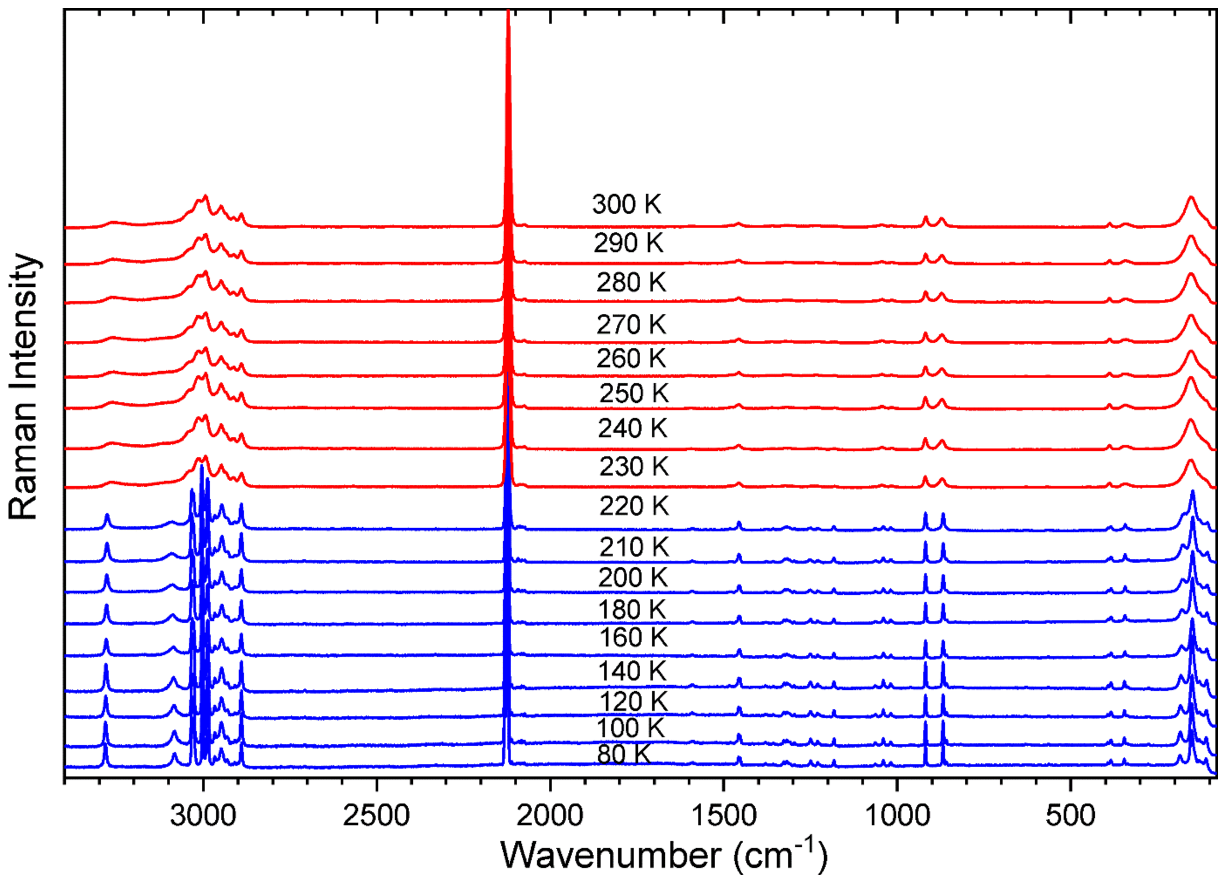

Figure S3. Temperature-dependent Raman spectra of $Pyr_2KCr(CN)_6$ in the 3400-80 cm^{-1} range.

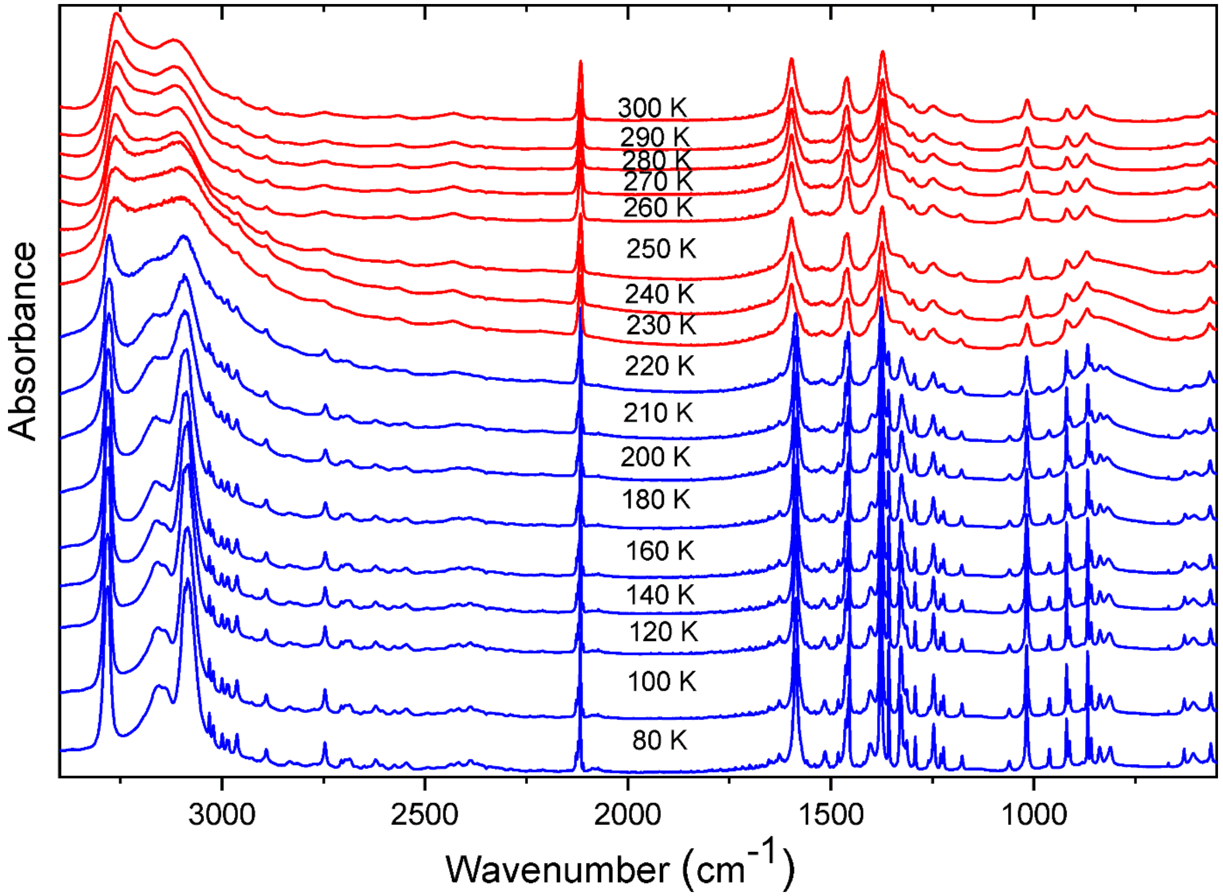

Figure S4. Temperature-dependent IR spectra of $Pyr_2KCr(CN)_6$ in the 3400-550 cm^{-1} range.

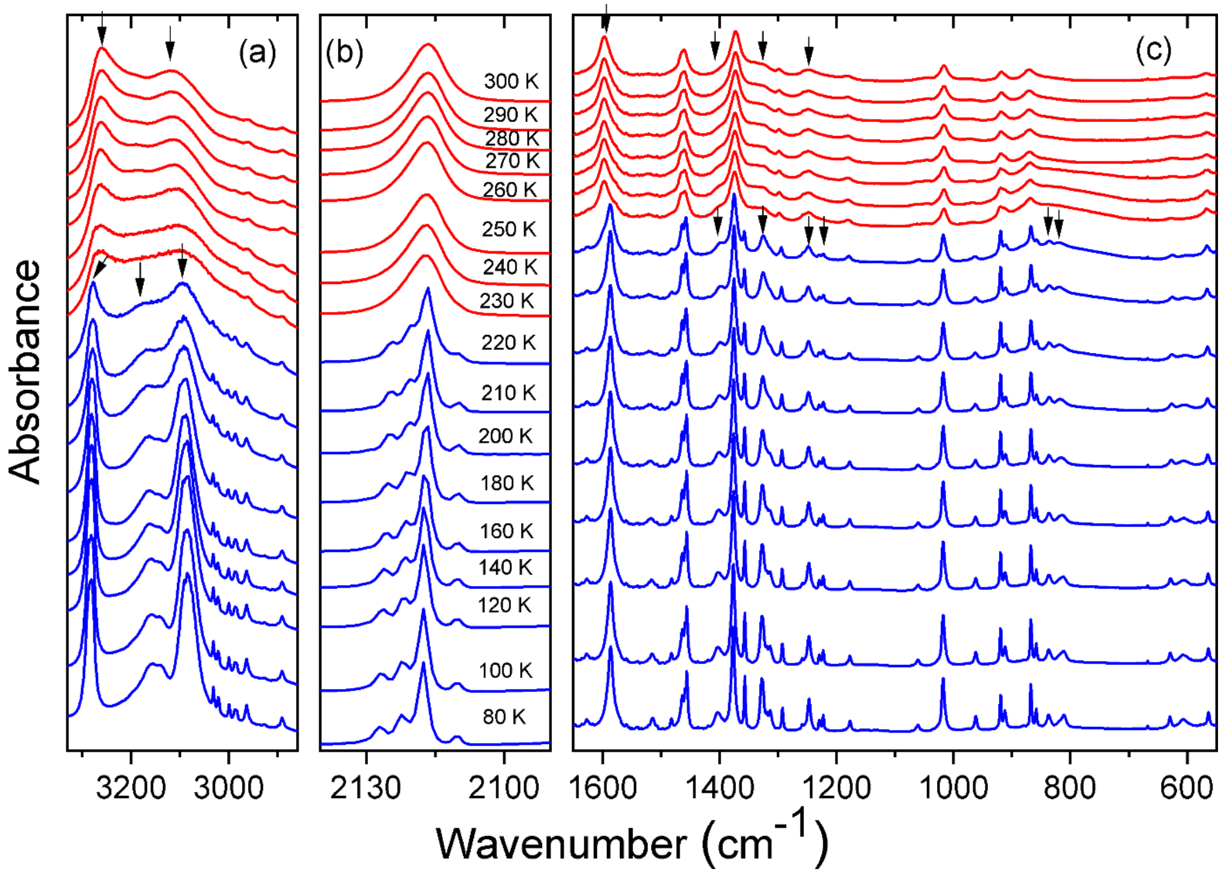

Figure S5. Temperature-dependent IR spectra of $Pyr_2KCr(CN)_6$ in the (a) 3330-2860 cm^{-1}, (b) 2140-2090 cm^{-1} and (c) 1650-550 cm^{-1} ranges. Arrows indicate modes related to vibrations of the NH_2 group.

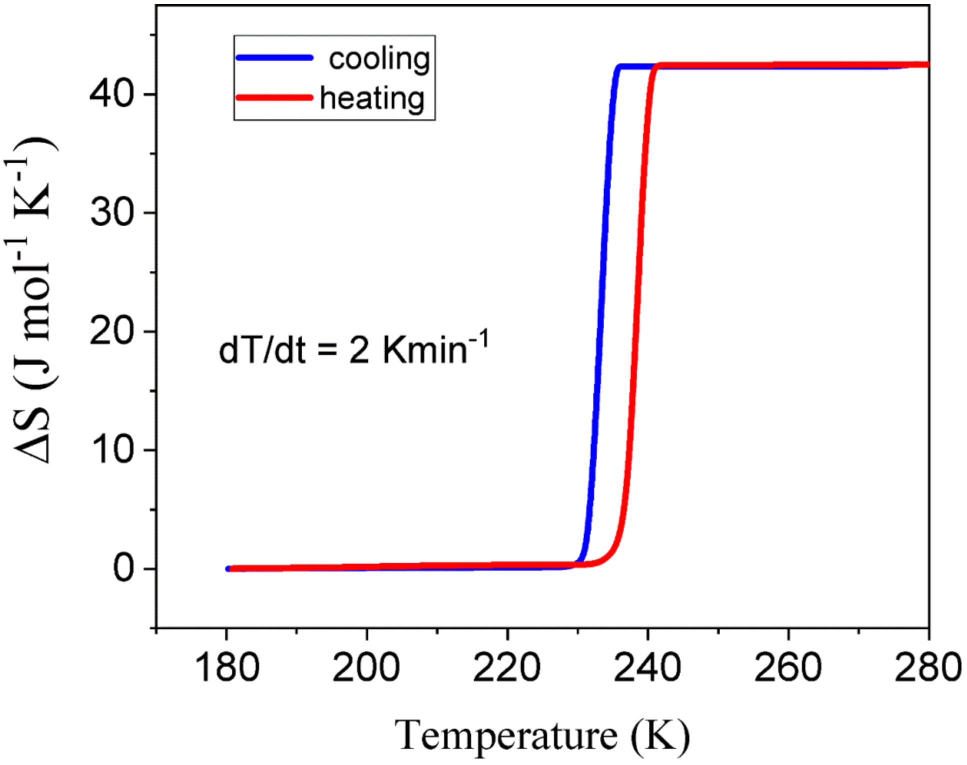

Figure S6. Change in entropy related to the phase transition in the heating (red) and cooling (blue) runs for $Pyr_2KCr(CN)_6$.

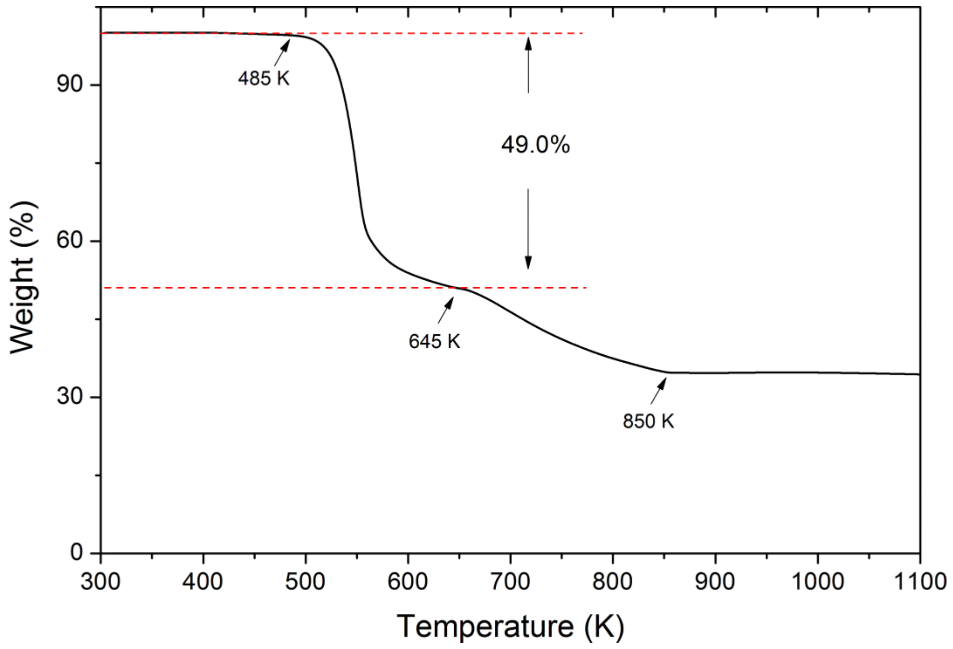

Figure S7. TGA data for $Pyr_2KCr(CN)_6$ measured in the range from 300K to 1100K.

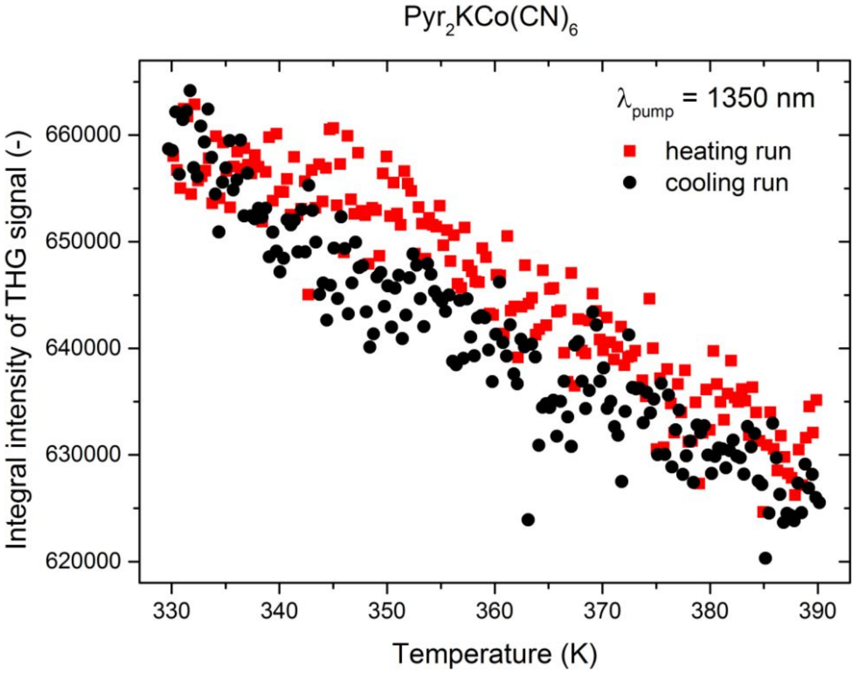

Figure S8. Plot of integral intensities of THG signal for $Pyr_2KCo(CN)_6$ measured at heating/cooling rate of 5 Kmin^{-1}. Note virtually no change of THG response at the phase transition temperature, as opposed to $Pyr_2KCr(CN)_6$.

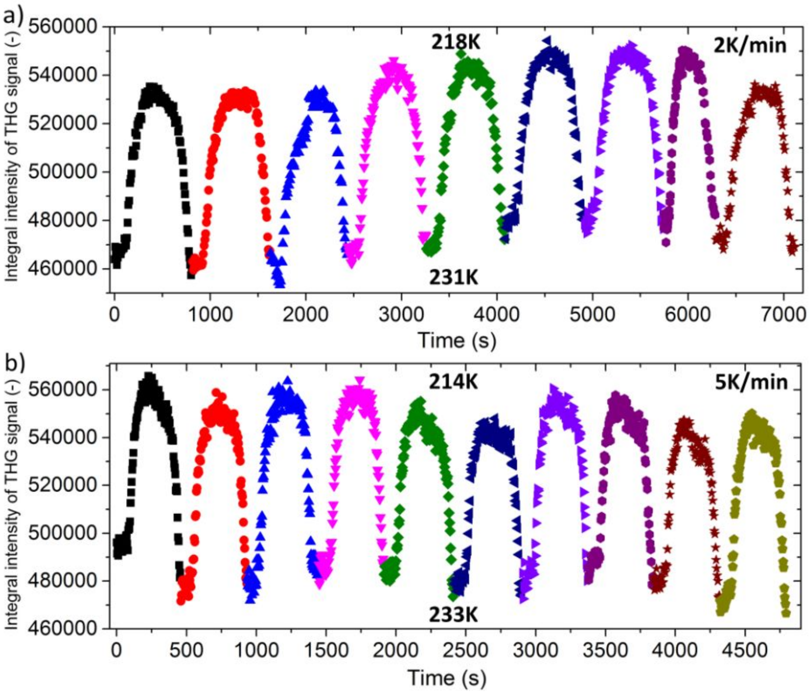

Figure S9. Plots of integral intensities of THG signal for $Pyr_2KCr(CN)_6$ obtained during switching experiment between for a heating/cooling rate of a) 2 $Kmin^{-1}$ and b) 5 $Kmin^{-1}$. Consecutive cycles are drawn with different colors. λ_{pump} = 1350nm.

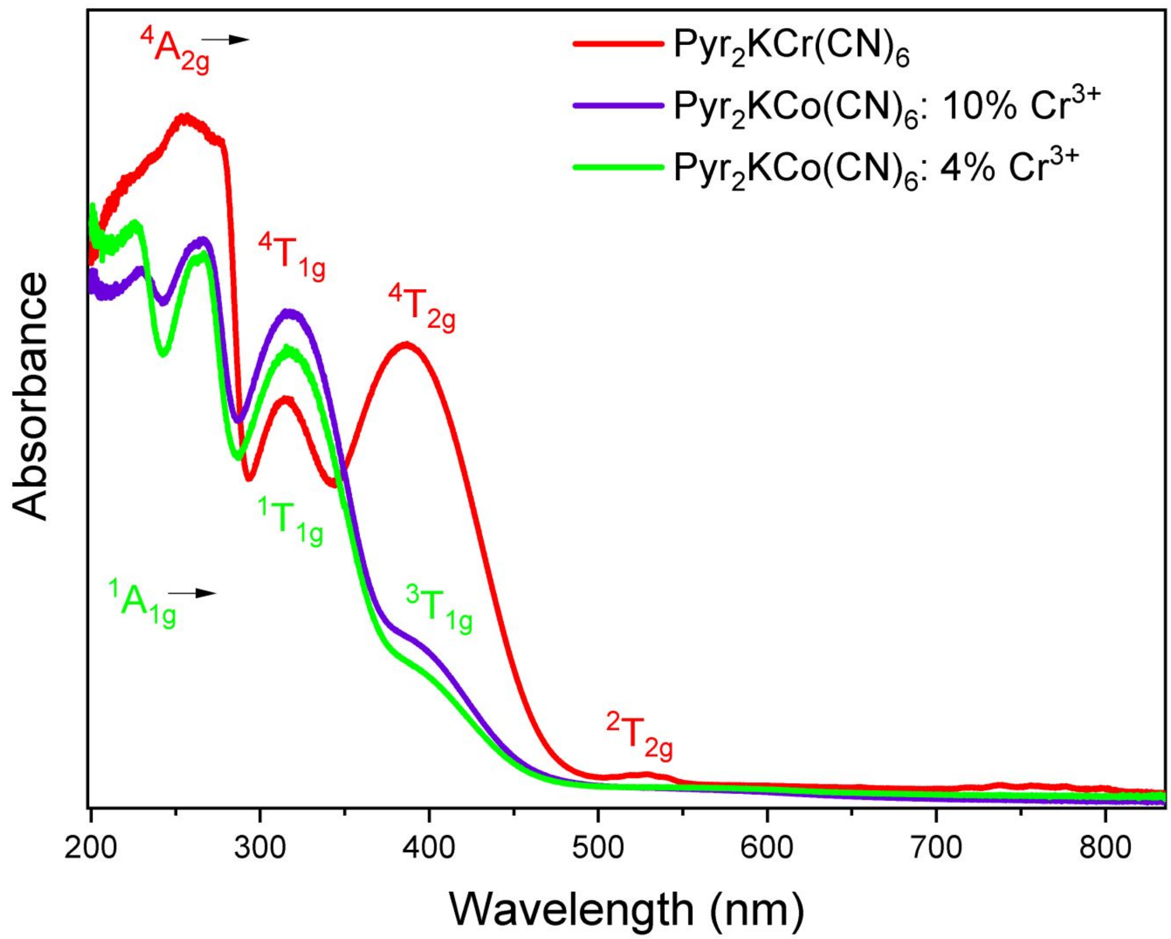

Figure S10. Room temperature absorption spectra of $Pyr_2KCr(CN)_6$ and $Pyr_2KCo(CN)_6$ doped with 4% and 10% of Cr^{3+} ions.

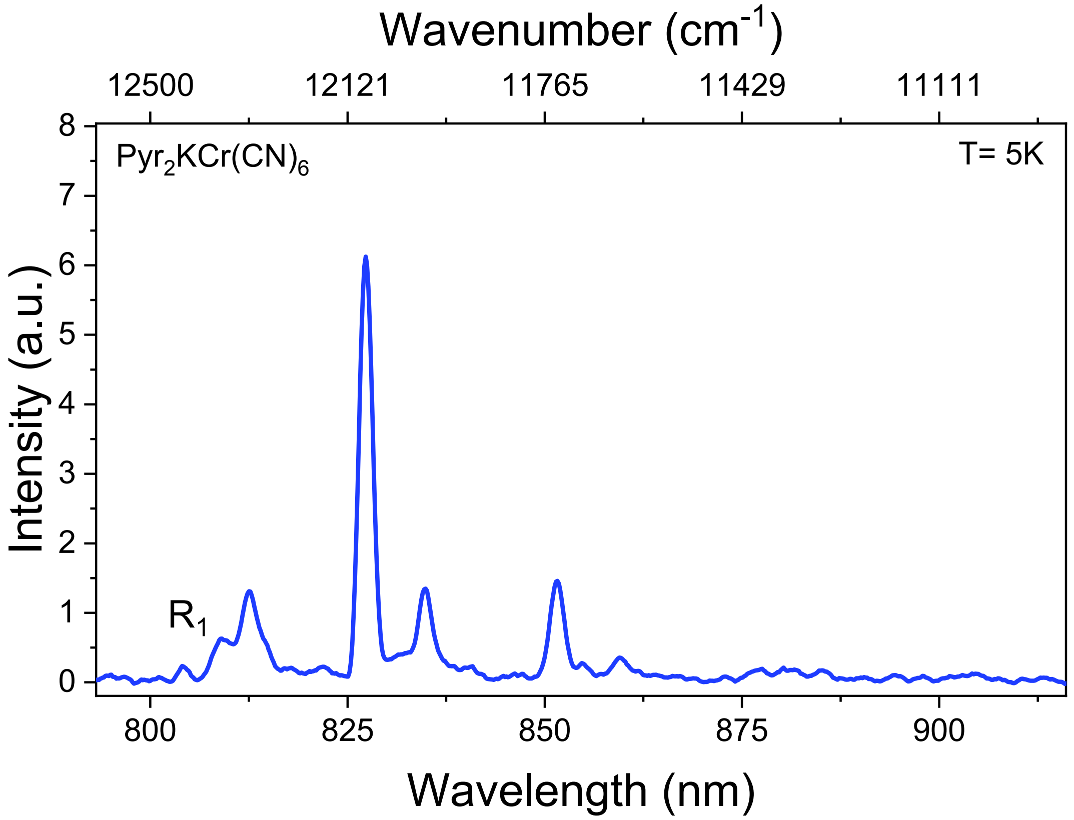

Figure S11. Low temperature emission spectra of the $Pyr_2KCr(CN)_6$ recorded at 5K.

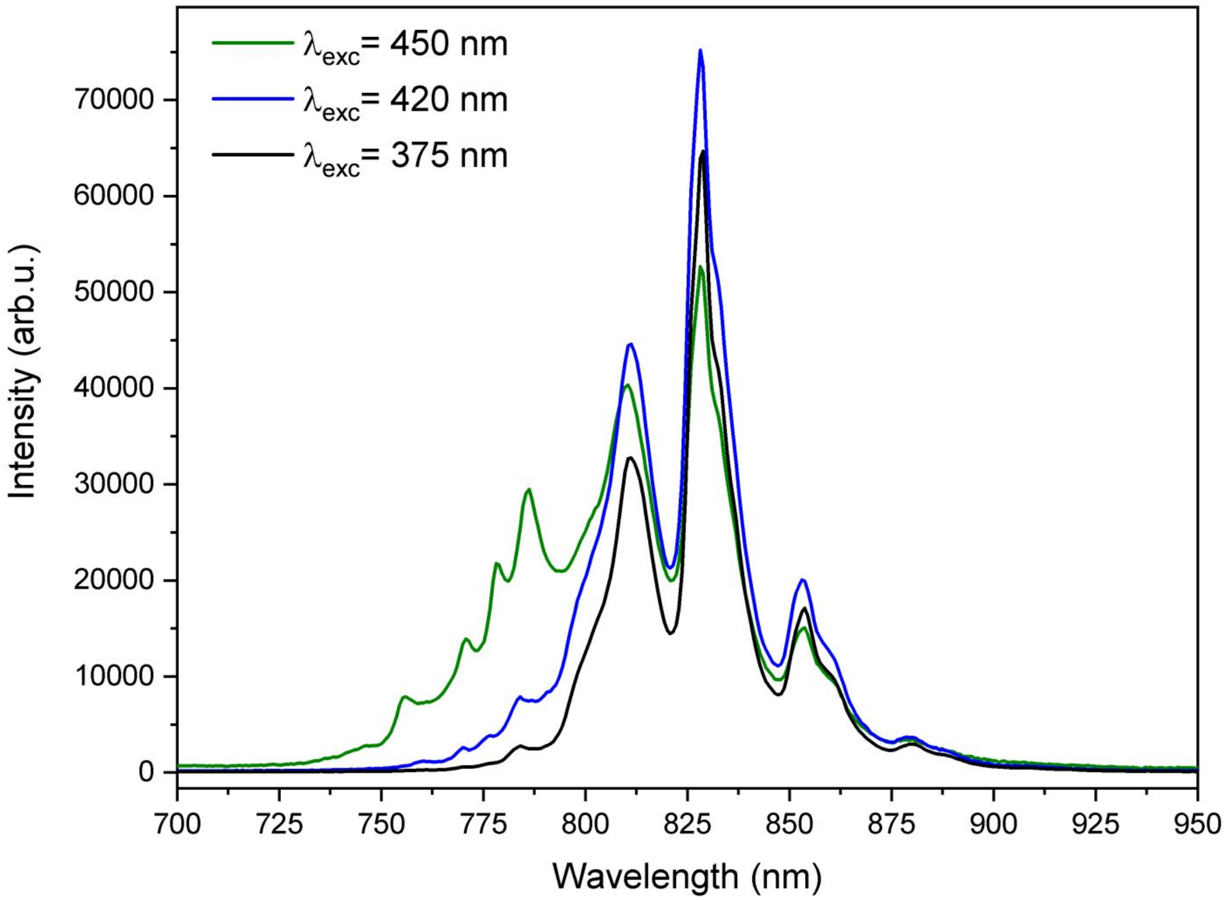

Figure S12. PL spectra of representative sample $Pyr_2KCo(CN)_6$: 4% Cr^{3+} recorded at 77 K under under various excitation lines.

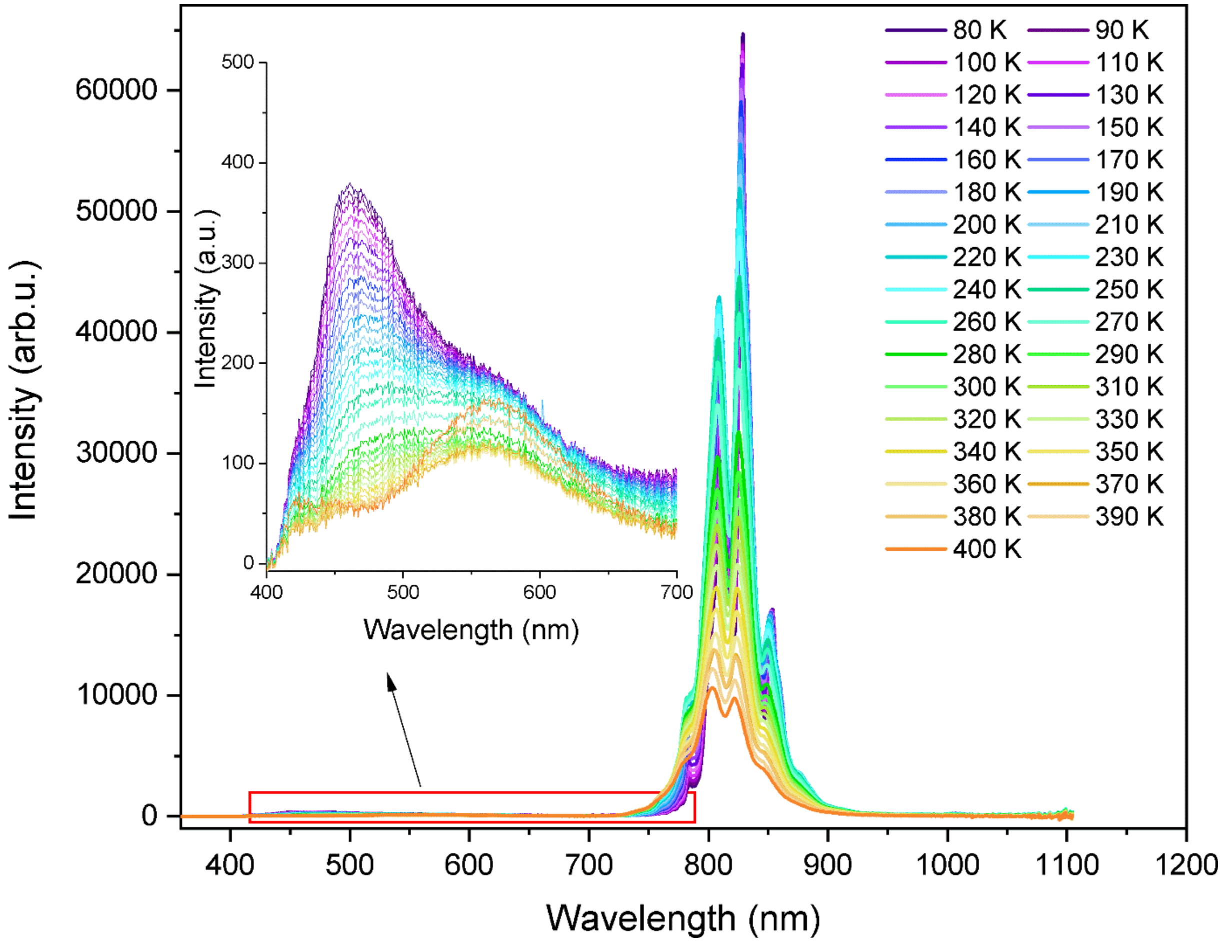

Figure S13. Temperature dependent emission spectra of $Pyr_2KCo(CN)_6$: 4% Cr^{3+} sample under 266 nm excitation.

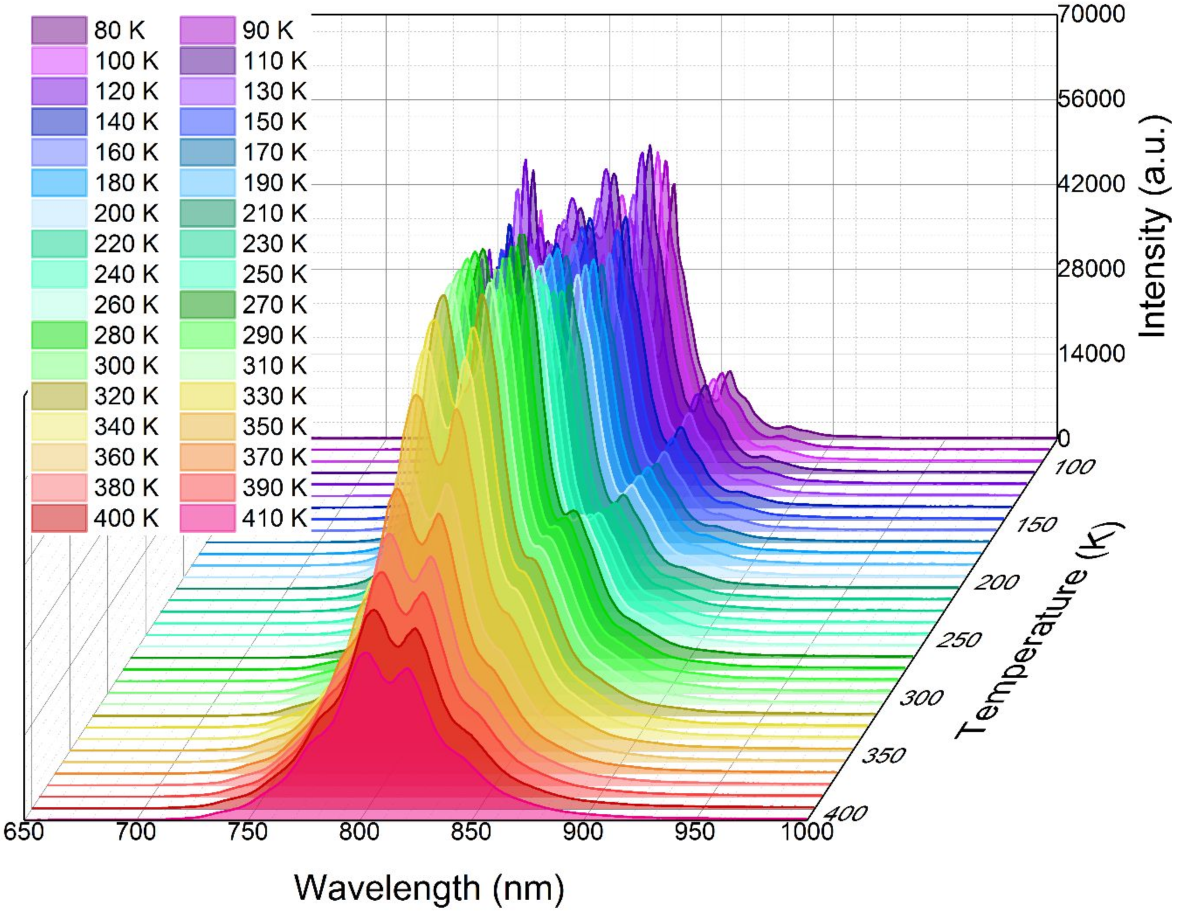

Figure S14. Temperature dependent emission spectra of $Pyr_2KCo(CN)_6$: 10% Cr^{3+} sample under 266 nm excitation.
